# Identification and functional prediction of long non-coding RNAs related to skeletal muscle development in Duroc pigs

**DOI:** 10.5713/ab.22.0020

**Published:** 2022-04-30

**Authors:** Lixia Ma, Ming Qin, Yulun Zhang, Hui Xue, Shiyin Li, Wei Chen, Yongqing Zeng

**Affiliations:** 1 Shandong Provincial Key Laboratory of Animal Biotechnology and Disease Control and Prevention, College of Animal Science and Technology, Shandong Agricultural University, Tai’an, Shandong 271018, China; 2 Shandong Ding Tai Animal Husbandry Co. Ltd., Jinan, Shandong 250300, China

**Keywords:** Duroc Pigs, Long Non-coding RNAs, mRNAs, Skeletal Muscle

## Abstract

**Objective:**

The growth of pigs involves multiple regulatory mechanisms, and modern molecular breeding techniques can be used to understand the skeletal muscle growth and development to promote the selection process of pigs. This study aims to explore candidate lncRNAs and mRNAs related to skeletal muscle growth and development among Duroc pigs with different average daily gain (ADG).

**Methods:**

A total of 8 pigs were selected and divided into two groups: H group (high-ADG) and L group (low-ADG). And followed by whole transcriptome sequencing to identify differentially expressed (DE) lncRNAs and mRNAs.

**Results:**

In RNA-seq, 703 DE mRNAs (263 up-regulated and 440 down-regulated) and 74 DE lncRNAs (45 up-regulated and 29 down-regulated) were identified. In addition, 1,418 Transcription factors (TFs) were found. Compared with mRNAs, lncRNAs had fewer exons, shorter transcript length and open reading frame length. DE mRNAs and DE lncRNAs can form 417 lncRNA-mRNA pairs (antisense, cis and trans). DE mRNAs and target genes of lncRNAs were enriched in cellular processes, biological regulation, and regulation of biological processes. In addition, quantitative trait locus (QTL) analysis was used to detect the functions of DE mRNAs and lncRNAs, the most of DE mRNAs and target genes of lncRNAs were enriched in QTLs related to growth traits and skeletal muscle development. In single-nucleotide polymorphism/insertion-deletion (SNP/INDEL) analysis, 1,081,182 SNP and 131,721 INDEL were found, and transition was more than transversion. Over 60% of percentage were skipped exon events among alternative splicing events.

**Conclusion:**

The results showed that different ADG among Duroc pigs with the same diet maybe due to the DE mRNAs and DE lncRNAs related to skeletal muscle growth and development.

## INTRODUCTION

Skeletal muscle occupies 45% to 60% of the animal body weight, it is the most abundant tissue in animals, and it is also one of the most important production traits for the growth and development of livestock and poultry animals. In medicine, abnormal regulation of skeletal muscle can lead many different types of muscle diseases, such as, myosarcoma, muscular atrophy, dystrophy, and muscular hypertrophy [1]. Failure of skeletal muscle development before birth can lead to embryonic death, and failure to repair or maintain skeletal muscle after birth can lead to a decline in the quality of life, and sometimes even death [2]. Therefore, exploring the mechanisms of skeletal muscle growth and development will help improve livestock production performance. High-throughput sequencing technology, genome-wide association study (WGAS), whole genome bisulfite sequencing (WGBS) and RNA-sequencing (RNA-seq) are gradually being applied to the study of skeletal muscle [3–5], and a large number of key candidate genes related to the growth and development of skeletal muscle have been identified [6,7].

The length of lncRNA is more than 200 nt, and the most of lncRNAs are transcribed by polymerase II, modified by splicing, capping, and tailing, and have characteristics similar to mRNA, including 5′cap and 3′polyA tail structure [8]. But compared with mRNA, it has poor conservation, low expression abundance and tissue specificity [9]. In recent years, with the continuous deepening of lncRNA function research, more and more studies have proved that lncRNAs can regulate muscle growth and differentiation, cell cycle and cell apoptosis [10,11]. lncRNA-Six1 exerts cis-regulation on Six1 gene encoding protein, and encodes micropeptide to active Six1 genes, thereby promoting cell proliferation and participating in muscle growth [12]. lncRNA-Dum is in skeletal myoblast cells, its expression is dynamically regulated during myogenesis. It is also transcriptionally introduced by MyoD binding during myoblast differentiation. The results show that it can promote myoblast differentiation and damage-induced muscle regeneration [13]. lnc133b can regulate bovine skeletal muscle satellite cell proliferation and cell apoptosis by mediating “sponge” miR-133b [14]. But RNA-seq is rarely applied to studies of full or half-siblings, this study may provide a novel method and clarify the relative precise mechanisms.

Duroc pigs are famous for their high growth rate, feed conversion efficiency, and lean meat percentage [15,16]. Duroc, as a typical lean pig breed, has good growth and meat production performance before 110 kg body weight, but its growth and meat production performance gradually decreases after 110 kg body weight. Therefore, the selection of 110 to 130 kg body weight can continue to maintain good growth and meat production performance, that is, cultivating a new breed of high-yield and high-quality meat pigs with large body size, which would have a great effect on improving the economic benefits of the pig industry. Therefore, this study aims to explore candidate lncRNAs and mRNAs related to skeletal muscle growth and development among Duroc pigs with different average daily gain (ADG).

## MATERIALS AND METHODS

### Ethics statement

All animal care and treatment procedures were conducted in strict accordance with the Animal Ethics Committee of Shandong Agricultural University, China, and performed in accordance with the Committee’s guidelines and regulations (Approval No.: 2004006).

### Animals

Duroc pigs came from a core breeding farm, with the measurement data in the pig herd to 30 to 110 kg body weight (individuals in the top 30% of ADG), and the performance measurement was continued to about 130 kg body weight. According to the ADG, 8 pigs were selected and divided into two groups: 4 high-ADG group (774.89 g) (H group) and 4 low-ADG group (658.77 g) (L group), and each pair of high and low groups were half siblings. The longissimus dorsi muscle (LDM) tissues were sampled and snap-frozen in liquid nitrogen for extraction of total RNA.

### RNA extraction, strand-specific library construction and sequencing

Total RNA was extracted from LDM tissues with Trizol reagent kit (Invitrogen, Carlsbad, CA, USA) according to the instructions. RNA quality (Supplementary Figure S5) was assessed on an Agilent 2100 Bioanalyzer (Agilent Technologies, Palo Alto, CA, USA), and checked with RNase free agarose gel electrophoresis. The enriched mRNAs and ncRNAs were fragmented into short fragments with fragmentation buffer after ribosome RNA (rRNA) removed. First-strand was transcribed with random primers. Second-strand of cDNA was synthesized with DNA polymerase I, RNase H, dUTP and buffer. Then, the cDNA was purified with QiaQuick polymerase chain reaction (PCR) extraction kit (Qiagen, Venlo, The Netherlands), end repaired, poly(A) added, and ligated to Illumina sequencing adapters. Then the second-strand cDNA was digested with uracil-N-glycosylase. The digested products were size selected with agarose gel electrophoresis, amplified, and sequenced with Illumina HiSeqTM 4000 (In this study, the paired-end sequencing method of Illumina sequencing was used, and each end was the 150 bp reads inserted into the target sequence, which were sequenced.) by Gene Denovo Biotechnology Co. (Guangzhou, China).

### Quality control for raw reads (raw datas) and mapping

Raw reads contained adapters or low quality reads were contained. Thus, reads were further filtered by using fastp [17] (https://github.com/OpenGene/fastp) (version 0.18.0) to get high quality clean reads. The quality control standards were as follows: i) removing reads with adapters; ii) removing reads with more than 10% of unknown nucleotides (N); iii) removing low quality reads with more than 50% of low quality (Q-value≤20) bases. Bowtie2 [18] (version 2.2.8) was used for mapping reads to rRNA database, the mapped reads were removed, and the remaining reads were further used in assembly and analysis of transcriptome. The rRNA removed reads of each sample were then mapped to reference genome by HISTA2 [19], respectively. After aligned with reference genome, on average about 84.10% clean reads were mapped to the reference genome (Ensembl-release104), and 77.60% clean reads were unique mapped, and only 6.5% clean reads were multiple mapped (Supplementary Table S1).

### Quantification and differentially expressed transcripts analysis

Transcript abundances were quantified with StringTie software in a reference-based approach. A FPKM (fragment per kilobase of transcript per million mapped reads) value was calculated to quantify. mRNAs and lncRNAs differential expression analyses were performed with DESeq2 software. The genes with the parameter of p-value below 0.05 and absolute fold change ≥1.5 were considered differentially expressed (DE) mRNAs and lncRNAs.

### Exon and intron analysis

The information of introns and exons exists in the structural annotation of the reference genome, so when the transcriptome data is compared with the reference genome, the intron and exon information of the compared genes can be directly called. When reads were aligned with introns, they belong to introns, and when they were aligned with exons, they belong to exons.

### Gene ontology and Kyoto encyclopedia of genes and genomes enrichment analysis

Gene ontology (GO) is an international standardized gene functional classification system, which includes three parts: cellular component (CC), biological process (BP), and molecular function. The analysis of Pathway helps to further know about the biological functions of genes. Kyoto encyclopedia of genes and genomes (KEGG) is the main public database about Pathway [20].

### lncRNA-mRNA association analysis

To reveal the interaction between antisense lncRNAs and mRNAs, the software RNAplex [21] (version 0.2) was used to predict the complementary binding between antisense lncRNAs and mRNAs. The ViennaRNA package is a program in RNAplex software [22].

One of the functions of lncRNAs is cis-regulation of their neighboring genes on the same allele. The up-stream lncRNAs which intersect a promoter or other cis-elements may regulate gene expression in transcriptional or post-transcriptional level. The down-stream or 3′UTR region lncRNAs may have other regulatory functions. Thus, lncRNAs which had been previously annotated as “unknown region” were annotated again. lncRNAs in less than 100 kb up/down stream of a gene were likely to be cis-regulators.

Another function of lncRNAs is trans-regulation of co-expressed genes not adjacent to lncRNAs. We analyzed the correlation of expression between lncRNAs and protein-coding genes to identify target genes of lncRNAs.

### Single-nucleotide polymorphism and insertion-deletion analysis

The GATK [23] (version 3.4–46) was used for calling variants of transcripts, and ANNOVAR [24] was used for single-nucleotide polymorphism/insertion-deletion (SNP/InDel) annotation. The following criteria were used to screen reliable editing sites from SNP sites: i) Removing the low quality SNP by GATK. ii) Correcting the SNP around INDEL region. iii) Choosing non-overlapping SNP in EXON and UTR region. iv) Choosing SNP with reference reads≥2 and variant reads ≥3. v) Choosing SNP with the variation frequency between 0.1 and 0.9 [25,26].

### Alternative splicing (AS) analysis

rMATS [27] (version 4.0.1) (http://rnaseq-mats.sourceforge.net/index.html) was used to identify alternative splicing (AS) events and analyze differential AS events. There are two methods to analyze AS by rMATS. Junction count (JC) only considered the number of splicing events spanning a splice site. Reads On Target and Junction Count (JCEC) considered not only the reads spanning the splice site, but also the number of splicing events for the reads that do not span the splice site (exon counts). At the same time, to count AS events matched to exonic regions, two methods were used to analyze AS events. AS events with a false discovery rate <0.05 in a comparison as significant AS events.

### Quantitative real-time polymerase chain reaction analysis

The total RNA was reverse-transcribed into cDNA by PrimeScript RT reagent kit (TaKaRa, Dalian, China), and the analyzed by SYBR Green Pro Taq HS Premix (Accurate Biotechnology (Hunan) Co., Ltd, Changsha, China). Primers were compounded by Sangon Biotech (Shanghai, China), and sequences of primers are shown in Table 1. β-Actin was used as a housekeeping gene. The fold change in expression was the obtained by 2^−ΔΔCT^ method, ΔΔCT = (CT_Target gene_ − CT_β-actin_)_H group_ − (CT_Target gene_ − CT_β-actin_)_L group_.

## RESULTS

### Identification of DE mRNAs and DE lncRNAs

**Figure 1:**
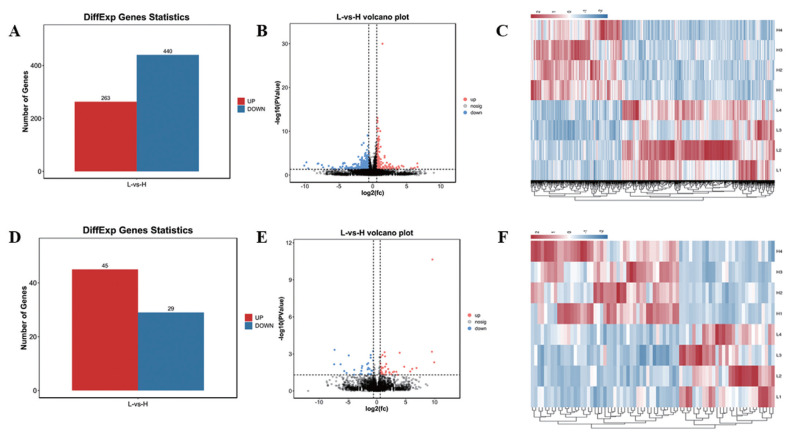
Statistics of DE mRNAs and DE lncRNAs. (A), (D) Statistics of 703 DE mRNAs and 74 DE lncRNAs. Red represents up-regulation, blue represents down-regulation. (B), (E) Volcano plot analysis of 703 DE mRNAs and 74 DE lncRNAs. Red represents up-regulation, blue represents down-regulation. (C), (F) Heat map analysis of 703 DE mRNAs and 74 DE lncRNAs. Each row represents a sample, and each column represents a DE mRNA or DE lncRNA. Red and blue gradients indicate increase and decrease in gene expression abundance, respectively. DE, differentially expressed.

In this study, 21,353 mRNAs were identified, including 61 novel genes and 21,292 known genes. 1,418 transcription factors (TFs) were found, the largest number is zf-C2H2 family (493 genes), 195 TFs belong to OTX family (Supplementary Figure S1). A total of 703 DE mRNAs were identified, including 263 up-regulated genes and 440 down-regulated genes (Figure 1A, B; Supplementary Table S9). In addition, a total of 10,178 lncRNAs were identified, including 8,478 Intergenic lncRNAs (Supplementary Figure S2A), and 479 lncRNAs were Scaffold, and the rest were located on the known chromosomes of pigs. This result showed that the distribution of the identified lncRNAs on the chromosomes did not have obvious bias (Supplementary Figure S2B). Compared with mRNAs, the average number of lncRNAs exon was 2.88, which was much lower than the number of mRNAs (9 exons) (Supplementary Figure S3A,B). The average length of mRNAs open reading frame (ORF) was 1,762 aa (Supplementary Figure S3D). The length of lncRNAs (2,747 nt) was shorter than mRNA (3,442 nt) (Supplementary Figure S3E, 3F). In addition, the relative expression level of lncRNAs was lower than mRNA (Supplementary Figure S3G, H). A total of 74 DE lncRNAs were identified, including 45 up-regulated lncRNAs and 29 down-regulated lncRNAs (Figure 1D, E; Supplementary Table S8). Hierarchical clustering showed that the expressions of genes and lncRNAs were distinguishable between H group and L group, indicating a significant difference between H group and L group (Figure 1C, 1F).

**Figure 2:**
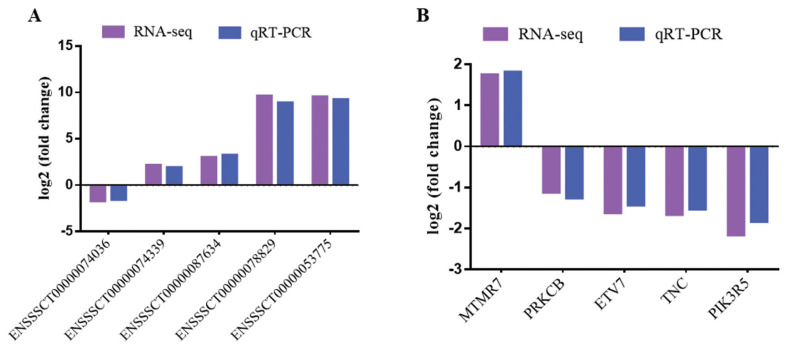
Validation of DE lncRNAs and DE mRNAs. (A) DE lncRNAs. (B) DE mRNAs. Purple represents RNA-seq result, blue represents qRT-PCR result. DE, differentially expressed; qRT-PCR, quantitative real-time polymerase chain reaction.

To verify RNA-seq results, five DE mRNAs and five lncRNAs were selected to perform by quantitative real-time polymerase chain reaction (qRT-PCR). The results showed that qRT-PCR results agreed with those in RNA-seq (Figure 2). The results indicated that DE mRNAs and DE lncRNAs identified from RNA-seq were reliable.

### lncRNA-mRNA association analysis

lncRNA-mRNA association analysis can be divided into three parts: antisense lncRNA analysis, cis regulation lncRNA analysis and trans regulation lncRNA analysis [28].

All lncRNAs/mRNAs and DE lncRNAs/mRNAs were analyzed (Supplementary Table S2). The results included one DE antisense lncRNA and one mRNA (Table 2).

One of the functions of lncRNAs is cis regulation of their neighboring genes on the same allele. As shown in Table 3, two DE lncRNAs and DE two mRNAs were identified (Table 3; Supplementary Table S3).

Another function of lncRNAs is trans regulation of co-expressed genes not adjacent to lncRNAs. Pearson correlation coefficient was used for samples≥6, and protein-coding genes with absolute correlation was more than 0.999. As shown in Table 4, 57 DE lncRNAs and 241 DE mRNAs were related, and 414 pairs lncRNA-mRNA were found.

### Gene ontology enrichment analyses of DE mRNAs and target genes of DE lncRNAs

**Figure 3:**
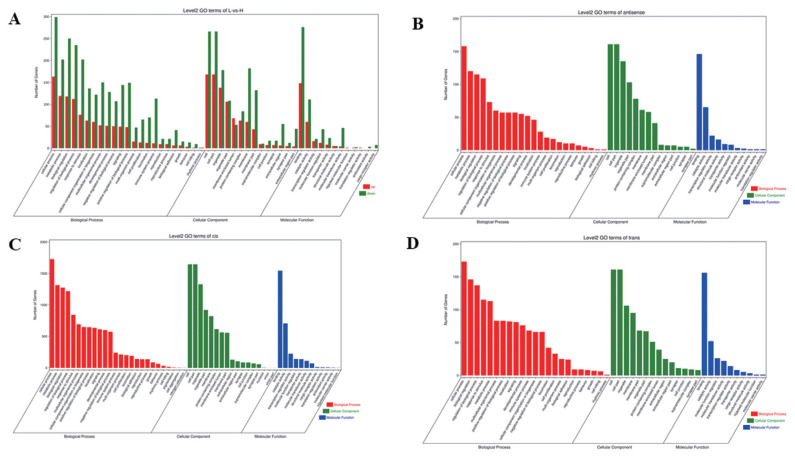
Analysis of GO enrichment. (A) DE mRNAs. The abscissa is the secondary GO term, the ordinate is the number of DE genes in this term, red means up-regulation, green means down-regulation. (B) Target genes of antisense regulation lncRNAs. (C) Target genes of cis regulation lncRNAs. (D) Target genes of trans regulation lncRNAs. (B), (C), (D) The abscissa is the secondary GO term, and the ordinate is the number of genes in this term. GO, gene ontology; DE, differentially expressed. Different colors show different types of GO terms, red represents biological process, green represents cellular component, blue represent molecular function.

To understand the functions of DE mRNAs and DE lncRNAs, the DE mRNAs and target genes of DE lncRNAs were annotated by GO enrichment analysis. The results showed that DE mRNAs were mainly enriched in BP, including cellular process, metabolic process, biological regulation, and regulation of BP. Cellular component was mainly including cell, cell part, organelle, and membrane part. Molecular function was mainly including binding, catalytic and molecular transducer activity (Figure 3A). The first 20 GO terms were mainly involved in biological process (Supplementary Figure S4A) (Q≤0.05). Target genes of lncRNAs (antisense, cis and trans regulation) were mainly enriched in cellular process, cell, cell parts and binding (Figure 3B, C, D). The first 20 GO terms were shown in Supplementary Figure S4B, S4C, S4D (Q≤0.05).

### KEGG analysis of DE mRNAs and target genes of DE lncRNAs

**Figure 4:**
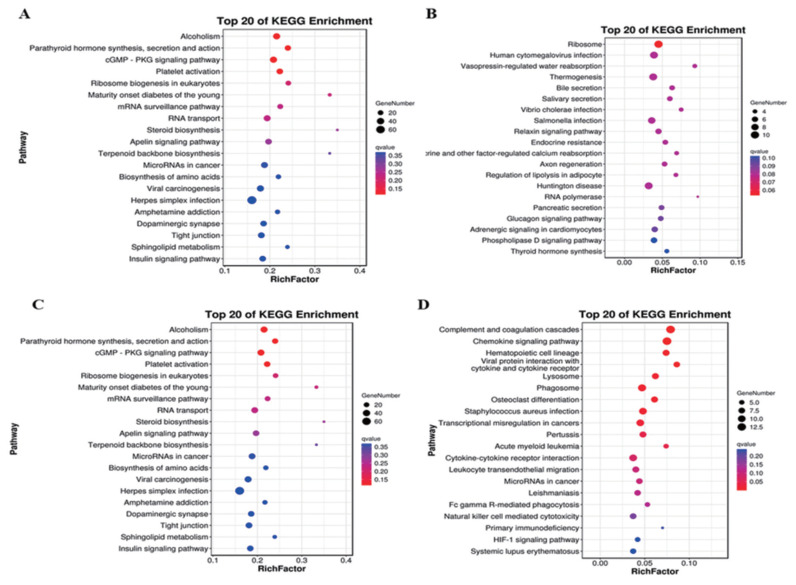
Analysis of KEGG enrichment. (A) DE mRNAs. (B) Target genes of antisense regulation lncRNAs. (C) Target genes of cis regulation lncRNAs. (D) Target genes of trans regulation lncRNAs. Using the top 20 pathways with the smallest Q value to make a map, the ordinate is the pathway, the abscissa is the enrichment factor, the size of the circle indicates the number of genes, the redder the color, the smaller the Q value. KEGG, Kyoto encyclopedia of genes and genomes; DE, differentially expressed.

A total of 703 DE mRNAs were enriched in 286 signaling pathways. Complement and coagulation cascades and viral protein interaction with cytokine and cytokine receptor signaling pathways were the two significantly pathways. These DE mRNAs were also significantly enriched in pathways including chemokine signaling pathway, mitogen-activated protein kinase (MAPK) cascade (p = 0.038), extracellular signal-regulated kinase 1 (ERK1) and ERK2 cascade (p = 0.003) and positive regulation of ERK1 and ERK2 cascade (p = 0.015) (Figure 4A). Target genes of antisense regulation lncRNAs were mainly enriched in pathways including ribosome, huntington disease, thermogenesis, and human cytomegalovirus infection (Figure 4B). Target genes of cis regulation lncRNAs were mainly enriched in alcohol, cyclic guanosine monophosphate protein kinase G -protein kinase G (cGMP-PKG) signaling pathway, plate activation and RNA transport signaling pathway (Figure 4C). while Target genes of trans regulation lncRNAs were mainly enriched in complement and coagulation cascades, chemokine signaling pahway, phagosome and cytokine-cytokine receptor interaction signaling pathway (Figure 4D).

### Expression analysis of growth traits and meat quality-relevant QTLs

Skeletal muscle is closely related to pig growth and meat quality. To further study the potential functions of DE mRNAs and DE lncRNAs. Basic Local Alignment Search Tool (blast) comparison was performed on the quantitative trait locus (QTL) (<2 Mb) with high confidence related to pig growth and meat quality traits. If the transcript or QTL interval is more than 50% duplicated, the transcript is located on this QTL. According to the QTL mapping analysis, 652 DE mRNAs were enriched in the QTLs related to growth traits and meat quality, including protein kinase C beta (*PRKCB*) confirmed by qRT-PCR, was enriched in Number of muscle fibers per unit area QTL and Cholesterol level QTL (Table 5). A total of 967 target genes of DE lncRNAs were enriched in QTLs. CD9, as target gene of lncNRA *MSTRG.12078.1*, was enriched in Backfat at first rib QTL and Marbling QTL, and the length of QTL was over 2,321 MB (Table 6).

### Single-strand nucleotide polymorphism and insertion-deletion analysis

**Figure 5:**
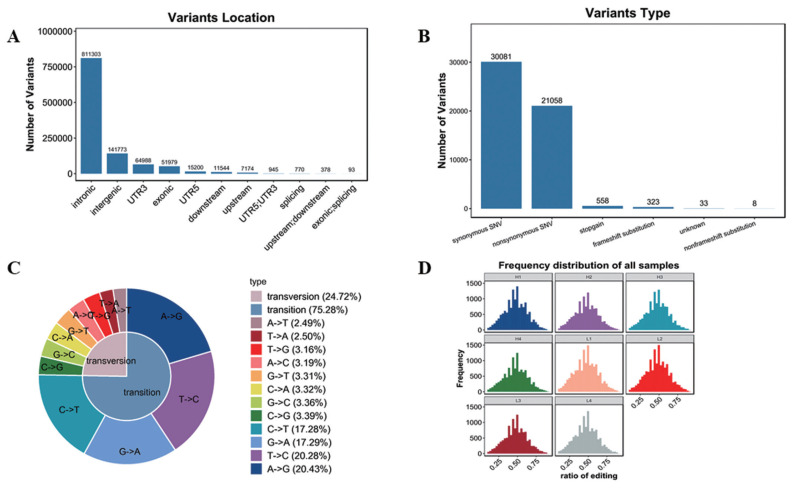
Analysis of variants. (A) Variants location. The abscissa represents variants location, the ordinate represents the number of variants. (B) Variants function. The abscissa represents variants type, the ordinate represents the number of variants. (C) Variants type. (D) Frequency distribution of all samples.

Variation analysis based on transcriptome sequencing mainly includes SNP and INDEL. In this study, 1,081,182 SNP and 131,721 INDEL were confirmed (Supplementary Table S4). As shown in Figure 5A, SNPs were mainly located in intronic and intergenic, in addition, SNPs in other locations were also found, such as 3′UTR, exonic, 5′UTR and other locations, but with far less than the intronic and intergenic SNPs. And functions were mainly synonymous single nucleotide variations (SNVs) and nonsynonymous SNVs (Figure 5B). SNP type includes transversion and transition. As shown in Figure 5C, the ratio of transversion was 24.72% (A-to-T 2.49%, T-to-A 2.50%, T-to-G 3.16%, A-to-C 3.19%, G-to-T 3.31%, C-to-A 3.32%, G-to-C 3.36%, C-to-G 3.39%), while transition percentage was 75.28% (C-to-T 17.28%, G-to-A 17.2-%, T-to-C 20.28%, A-to-G 20.43%).

RNA editing is a post transcriptional modification widely existing in eukaryotes. At present, SNVs are common in RNA editing. In this study, the frequency of SNVs was similar in 8 samples (Figure 5D, Supplementary Table S5).

### Alternative splicing analysis

**Figure 6:**
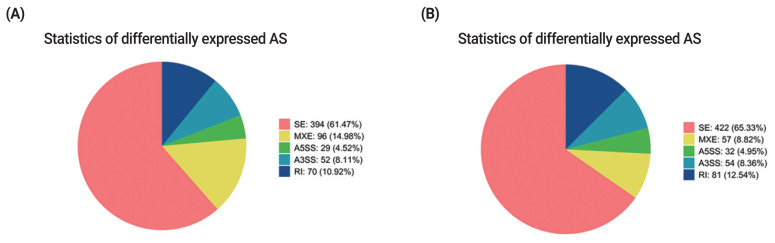
Statistics of differentially expressed AS events. (A) Statistics of differentially expressed AS events by JC. (B) Statistics of differentially expressed AS events by JCEC. Different colors show different types of AS events. AS, alternative splicing; JC, junction count; JCEC, reads on target and junction count.

Alternative splicing (AS) is an important regulation mechanism in eukaryotes. Five common types of AS events include skipped exon (SE), alternative 5′splice site (A5SS), alternative 3′splice site (A3SS), mutually exclusive exon (MXE), and retained intron (RI). AS events were analyzed by two methods (JC and JCEC). The results showed that the largest number of AS events was SE, followed by MXE (Supplementary Table S6, S7). Over 60% of differentially expressed AS events was SE, and the rest AS events accounted for less than 40% individually, suggesting that SE was the most common AS events in this study (Figure 6).

## DISCUSSION

In this study, Duroc pigs with different ADG were used for whole transcriptome sequencing to screen out the key lncRNAs and mRNAs that affect the later growth and development of large pigs. In recent years, studies have found that lncRNAs are widely involved in a variety of human diseases and other life processes, and they have become a hot spot in biological research. Similarly, lncRNAs also play an important regulatory role, which is closely related to the meat quality of livestock and poultry [14,29]. Because of low conservation of lncRNAs [30], therefore it is necessary to understand the genomic characteristics of lncRNAs. In this study, 10,178 lncRNAs and 21,353 mRNAs were identified from the eight LDM libraries, and the results also showed that the characteristics of exon number, length of ORF, length distribution of transcripts and genomic expression level of lncRNAs were lower than mRNAs, which were similar to the previous reports [31,32]. These lncRNAs maybe play a key role in growth trait and skeletal muscle development, therefore the specific function still needs to be further studied.

The biological function of DE genes can be explained to a certain content by QTL analysis. Genome information can be downloaded from AnimalQTLdb (PigQTLdb: http://www.animalgenome.org-/QTLdb/pig.html) Database. Over 92% DE mRNAs and Target genes of DE lncRNAs were enriched QTLs related to skeletal muscle development, meat quality and metabolism. For example, myocyte enhancer factor 2C (*MEF2C*) (up-regulated) and *UACA* (up-regulated) were enriched in percentage type I fibers QTL, diameter of type IIb muscle fibers QTL, drip loss QTL and ADG QTL. MEF2C proteins have the potential contributions to muscle regeneration [33]. And cell proliferation and invasion of hepatocellular carcinoma cells can be inhibited by knockdown *UACA* [34]. It has been shown that *TNC* is a member of the tenascin gene family, and it was first reported in glioblastomas [35]. It is reported that *TNC* expression can affect cell behavior in many ways, and *TNC* level can be used as a biomarker of colorectal cancer [36,37]. In this study, *TNC* was enriched in loin muscle area QTLs and other QTLs. *MYO1F* were enriched in percentage type IIa fibers QTLs, ADG QTLs, carcass length QTLs, body weight (end of test) QTLs and QTLs related to meat quality. These findings may provide a new method for studying the potential functions of these genes.

To explore the candidate genes related to skeletal muscle development, association analysis was performed between DE mRNAs and lncRNAs. In recent years, more and more research has found a key role of lncRNAs in skeletal muscle growth and development, and found that they are closely related to muscle diseases [2,38]. As shown in a report, highly expressed DE lncRNA *MSTRG.42019* in skeletal muscle, has a positive correlation with myosin heavy chain 7 (*MYH7*), and a negative correlation with meat quality traits [32]. LncRNA *linc-MD1* exerts functions by miR-133 and miR-135 [39]. Antisense lncRNA FOXF1 adjacent non-coding developmental regulatory RNA (*FOXF1-AS1*) can promote migration by the FOXF1/MMP-2/−9 pathway. The study detected trans lncRNA ENSSSCT00000046000-MMP9 pair, which was related to cancer. Therefore, it is necessary to study DE lncRNAs and mechanisms.

In GO and KEGG results, although the top 20 GO terms and pathways have no pathways related to skeletal muscle growth and development, some related pathways were still analyzed, which were identified to be related with skeletal muscle development [40,41]. In this study, it was found that antisense LncRNA MSTRG.6467.1-MTMR7 pair was related to inositol phosphate metabolism and phosphatidylinositol signaling system. The cis LncRNA MSTRG. 12078.1-CD9 were enriched in hematopoietic cell lineage. Although the specific function of the identified DE mRNAs and lncRNAs are unclear, it also provided some signaling pathways for the growth and development mechanism of skeletal muscle.

RNA editing can efficiently change the amino acid sequence of the protein, thereby changing the genetic information the genome template [42]. Abnormal RNA editing is closely related to human diseases such as amyotrophic lateral sclerosis and cancer [43]. The diversity of RNA in eukaryotes mainly comes from A-to-G. In this study, the frequency of transition (75.28%) is significantly higher than transversion (24.72%). And A-to-G was the most frequent, which was similar with previous research [44]. The diversification of RNA and DNA editing types was designed to the development of genetics, molecules, biochemistry, and other aspects to solve biological problems, but there are still many challenges. With the development of high-throughput technology, these problems will gradually be solved.

AS is a crucial factor in increasing the complexity of cell functions [45], the identified AS events will help to better know about the regulation mechanism skeletal muscle development. rMATS [46] was used to analyze AS. SE was the most common of the AS events, which was different from some reports [3,47]. This may be related to the different analysis methods and samples. As shown in a report, myotilin (*MYOT*) lack an entire exon13 [48], In addition, the AS events occurring in *MEF2C* and *MEF2D* have a certain regulation effect on skeletal muscle differentiation and myogenesis [45,49]. The AS events of *MEF2C*, *MEF2D*, and *MYOT* were found in this study, which can provide certain data support for the further study of genes related to skeletal muscle growth and development.

## CONCLUSION

In conclusion, 703 DE mRNAs and 74 DE lncRNAs were identified. Compared with mRNAs, lncRNAs had fewer exons, shorter transcript length and ORF length. DE mRNAs and DE lncRNAs can form 417 lncRNA-mRNA pairs (antisense, cis, trans). In addition to cell and cell parts, DE mRNAs and target genes of lncRNAs were also enriched in MAPK cascade and ERK1/ERK2 cascade. In addition, QTL analysis was used to detect the functions of DE mRNAs and lncRNAs, the most of DE mRNAs and target genes of lncRNAs were enriched in QTLs related to skeletal muscle development and meat quality. In SNP/INDEL analysis, 1,081,182 SNP and 131,721 INDEL were found, and transition was more than transversion. Over 60% of percentage were SE events among AS events. The large amount of data identified by RNA-seq in this study requires more technical verification to select high-quality pigs, and to further provide a theoretical basis for pig genetic improvement and meat quality research.

## Figures and Tables

**Table 1:** Primers sequences

Gene	Sequence (5′-3′)
lncRNA ENSSSCT00000053775-F	TTAGGTACGCCTCCCCAACTTCC
lncRNA ENSSSCT00000053775-R	GGCAACAGATTCCAGACCCAGATC
lncRNA ENSSSCT00000078829-F	CACCTGCCATTGTCCATCTCTGTC
lncRNAENSSSCT00000078829-R	CGGAGCTTCCAGACTAGGAGAGG
lncRNAENSSSCT00000087634-F	GCATTCCACAGACATCAGAGAGTCC
lncRNAENSSSCT00000087634-R	GTAGAAGCAGAGAAGGCAGCAAGG
lncRNAENSSSCT00000074339-F	CACAGCCACAGCCACAACAG
lncRNAENSSSCT00000074339-R	TAAGGATCCAGCGTTGCCGT
lncRNAENSSSCT00000074036-F	TGGAGACGCCCTTGACCAAG
lncRNAENSSSCT00000074036-R	GTGATGGGCTGGCTGGAGAT
*MTMR7*-F	AGACTCTTCACTCGGGACGGTAAC
*MTMR7*-R	GGTGGTGCAGGAGGAGTAGGAG
*PRKCB*-F	ATTCACCGCCCGCTTCTTCAAG
*PRKCB*-R	AACAGCAGACTTGGCACTGGAATC
*ETV7*-F	AAGAAGGAACCTGGGCAGAAACTC
*ETV7*-R	GTCTGTCCTGCTCCTGGCTCTC
*TNC*-F	CTGCCACCGAATACACGCTGAG
*TNC*-R	GCTGTTTCCGACTGAACCTCTGTAG
*β-actin*-F	CCAGGTCATCACCATCGGCAAC
*β-actin*-R	CAGCACCGTGTTGGCGTAGAG

*MTMR7*, myotubularin related protein 7; *PRKCB*, protein kinase C beta; *ETV7*, ETS variant transcription factor 7; *TNC*, tenascin C.

**Table 2:** Differentially expressed antisense regulation results

lncRNA_ID	mRNA_ID	Symbol	Description
*MSTRG.6467.1*	ENSSSCG00000036785	MTMR7	myotubularin related protein 7

**Table 3:** Differentially expressed cis regulation results

lncRNA_ID	mRNA_ID	Symbol	Description
*ENSSSCT00000087573*	ENSSSCT00000087573	-	-
*MSTRG.12078.1*	ENSSSCG00000022230	CD9	CD9 moleculeprotein 7

**Table 4:** Trans regulation results

List	lncRNA	mRNA	Pair
Diff	57	241	414

**Table 5:** Differentially expressed mRNAs distribution in chromosome and QTLs regions

Chromosome/mitochondrion	DE gene number	DE gene number in QTL region	QTL region length (Mb)
1	51	51	272.538
2	75	74	152.534
3	37	36	129.972
4	48	47	126.574
5	38	35	98.508
6	73	67	162.985
7	49	49	126.47
8	27	25	143.374
9	34	33	138.044
10	6	5	67.1919
11	9	9	77.1184
12	31	29	58.0079
13	51	51	208.259
14	46	42	154.596
15	25	24	137.024
16	18	17	66.7432
17	22	19	55.1875
18	21	21	58.1156
X	25	18	85.3869
Y	1	0	0
MT	8	0	0

DE, differentially expressed; QTL, quantitative trait locus.

**Table 6:** Target genes of DE lncRNAs distribution in chromosome and QTLs regions

Chromosome/Mitochondrion	DE gene number	DE gene number in QTL region	QTL region length (Mb)
1	95	95	272.538
2	91	89	152.534
3	59	58	129.972
4	64	60	127.273
5	62	56	98.508
6	108	104	162.985
7	68	68	128.937
8	45	40	143.428
9	47	47	138.044
10	21	20	67.1919
11	15	14	78.2374
12	49	45	58.0079
13	79	79	208.259
14	51	46	154.596
15	41	41	137.024
16	24	24	66.7432
17	38	34	55.1875
18	25	25	58.1156
X	29	22	83.7497
Y	4	0	0
MT	2	0	0

DE, differentially expressed; QTL, quantitative trait locus.

## SUPPLEMENTARY MATERIAL

The data used to support the findings this study is available from the corresponding author upon request.

Supplementary Table S1. Statistics for filtering and mapping reads

Supplementary Table S2. Analysis of antisense regulation lncRNAs

Supplementary Table S3. Analysis of cis regulation lncRNAs

Supplementary Table S4. Analysis of SNP/INDEL

Supplementary Table S5. SNVs distribution of all samples

Supplementary Table S6. Statistics of AS events by JC

Supplementary Table S7. Statistics of AS events by JCEC

Supplementary Table S8. The list of DE lncRNAs

Supplementary Table S9. The list of DE mRNAs

Supplementary Table S10. Analysis of traits

Supplementary Figure S1. Statistics of Transcription factors (TFs). The abscissa is the TF family, the ordinate is the number of genes.

Supplementary Figure S2. Analysis of lncRNA type and the chromosome distribution of identified lncRNAs.

Supplementary Figure S3. Comparison of genomic characteristics between lncRNAs and mRNAs.

Supplementary Figure S4. The first 20 GO terms.

Supplementary Figure S5. Electropherogram analysis of Total RNA on Agilent 2100 Bioanalyzer.
