# Supplementary material for: Identification and functional prediction of long non-coding RNAs related to skeletal muscle development in Duroc pigs
Source: Anim Biosci. 2022 Apr 30;35(10):1512–23. doi: 10.5713/ab.22.0020 (PMC9449383; doi:10.5713/ab.22.0020)
Supplement: Supplementary Table S1. — Statistics for filtering and mapping reads [file ab-22-0020-suppl1.pdf]

Table S1 Statistics for filtering and mapping reads

Sample	Total[a]	Unmapped[b]	Unique_ Mapped[c]	Multiple_ Mapped[d]	Total_ Mapped[e]
H1	59964760	9066482	47380876	3517402	50898278
H2	63533624	10671923	48784652	4077049	52861701
H3	56794110	9534115	43462525	3797470	47259995
H4	60582058	9378794	46859700	4343564	51203264
L1	63935382	10529588	49131821	4273973	53405794
L2	69215198	11304511	53523484	4387203	57910687
L3	63074270	9600083	49311578	4162609	53474187
L4	58074524	8705438	45735733	3633353	49369086

[a] Total: Total number of clean reads

[b] Unmapped: Reads not aligned to the reference sequence

[c] Unique_ Mapped: Reads aligned to only one position

[d] Multiple_ Mapped: Reads aligned to two or more positions

[e] T Total_ mapped: Reads that completely aligned to the reference sequence
