# Supplementary material for: Identification and functional prediction of long non-coding RNAs related to skeletal muscle development in Duroc pigs
Source: Anim Biosci. 2022 Apr 30;35(10):1512–23. doi: 10.5713/ab.22.0020 (PMC9449383; doi:10.5713/ab.22.0020)
Supplement: Supplementary Table S2. — Analysis of antisense regulation lncRNAs [file ab-22-0020-suppl2.pdf]

Table S2 Analysis of antisense regulation lncRNAs

List	lncRNA	mRNA	pair
All	234	234	259
Diff	1	1	1
