# Supplementary material for: Identification and functional prediction of long non-coding RNAs related to skeletal muscle development in Duroc pigs
Source: Anim Biosci. 2022 Apr 30;35(10):1512–23. doi: 10.5713/ab.22.0020 (PMC9449383; doi:10.5713/ab.22.0020)
Supplement: Supplementary Table S4. — Analysis of SNP/INDEL [file ab-22-0020-suppl4.pdf]

Table S4 Analysis of SNP/INDEL

Sample	All	SNP	INDEL
All	1212903	1081182	131721
H1	623657	556705	66952
H2	521806	467138	54668
H3	529271	473830	55441
H4	582435	520804	61631
L1	579693	518429	61264
L2	624697	558939	65758
L3	560668	501823	58845
L4	571473	510451	61022
