# Supplementary material for: Identification and functional prediction of long non-coding RNAs related to skeletal muscle development in Duroc pigs
Source: Anim Biosci. 2022 Apr 30;35(10):1512–23. doi: 10.5713/ab.22.0020 (PMC9449383; doi:10.5713/ab.22.0020)
Supplement: Supplementary Table S5. — SNVs distribution of all samples [file ab-22-0020-suppl5.pdf]

Table S5 SNVs distribution of all samples

Samples	H1	H2	H3	H4	L1	L2	L3	L4
All	14244	12456	13114	11981	15428	14993	12223	13355
A->T	239	229	215	190	234	249	210	218
A->C	399	320	336	321	437	441	327	374
A->G	2459	2200	2251	2022	2685	2630	2127	2253
T->A	229	190	197	173	221	211	181	205
T->C	2792	2426	2445	2299	2960	2792	2280	2606
T->G	333	309	323	301	249	348	303	333
C->A	414	342	349	368	463	443	355	375
C->T	3160	2818	3052	2732	3520	3286	2745	3009
C->G	511	415	482	411	535	546	435	484
G->A	2762	2413	2585	2360	2995	3043	2468	2626
G->T	388	306	352	326	412	430	314	338
G->C	558	488	527	478	617	574	478	534
