# Supplementary material for: Identification and functional prediction of long non-coding RNAs related to skeletal muscle development in Duroc pigs
Source: Anim Biosci. 2022 Apr 30;35(10):1512–23. doi: 10.5713/ab.22.0020 (PMC9449383; doi:10.5713/ab.22.0020)
Supplement: Supplementary Table S6. — Statistics of AS events by JC [file ab-22-0020-suppl6.pdf]

Table S6 Statistics of AS events by JC

AStype	known	novel
SE	4059	15104
MXE	483	2169
A5SS	722	277
A3SS	1140	384
RI	1496	69
