# Supplementary material for: Identification and functional prediction of long non-coding RNAs related to skeletal muscle development in Duroc pigs
Source: Anim Biosci. 2022 Apr 30;35(10):1512–23. doi: 10.5713/ab.22.0020 (PMC9449383; doi:10.5713/ab.22.0020)
Supplement: Supplementary Table S7. — Statistics of AS events by JCEC [file ab-22-0020-suppl7.pdf]

Table S7 Statistics of AS events by JCEC

AStype	known	novel
SE	4130	15207
MXE	489	2174
A5SS	729	280
A3SS	1147	384
RI	1521	71
