# Supplementary material for: Identification and functional prediction of long non-coding RNAs related to skeletal muscle development in Duroc pigs
Source: Anim Biosci. 2022 Apr 30;35(10):1512–23. doi: 10.5713/ab.22.0020 (PMC9449383; doi:10.5713/ab.22.0020)
Supplement: Supplementary Table S8. — The list of DE lncRNAs [file ab-22-0020-suppl8.pdf]

Table S8 The list of DE lncRNAs

Sus scrofa Ensemble ID	log2(fc)	PValue	FDR
ENSSSCT00000078829	9.634811	2.28E-11	1.02E-07
ENSSSCT00000087356	-7.32193	0.000464	0.601687
MSTRG.6871.1	-0.65707	0.000605	0.601687
ENSSSCT00000053775	9.54593	0.000669	0.601687
MSTRG.12068.1	1.342575	0.000728	0.601687
ENSSSCT00000079762	3.958421	0.000804	0.601687
MSTRG.621.1	0.97904	0.001238	0.648298
MSTRG.13674.2	-1.05856	0.00124	0.648298
ENSSSCT00000071964	-4.85798	0.00134	0.648298
MSTRG.4059.2	1.420751	0.001577	0.648298
MSTRG.575.2	0.674086	0.001756	0.648298
MSTRG.2959.3	-1.0663	0.002022	0.648298
MSTRG.6314.3	-0.63494	0.003354	0.997822
MSTRG.15785.1	9.940314	0.004856	0.997822
MSTRG.14363.3	-1.48543	0.005551	0.997822
MSTRG.2781.1	0.619605	0.00592	0.997822
MSTRG.6467.1	1.628355	0.006515	0.997822
ENSSSCT00000074036	-1.68544	0.00684	0.997822
ENSSSCT00000070306	-6.27612	0.006944	0.997822
ENSSSCT00000081165	-7.38802	0.006969	0.997822
ENSSSCT00000046000	-2.53811	0.0096	0.997822
MSTRG.12078.1	1.429744	0.010738	0.997822
ENSSSCT00000082572	4.754888	0.010954	0.997822
MSTRG.17089.1	0.809824	0.011448	0.997822
MSTRG.5126.3	0.804756	0.011828	0.997822
MSTRG.6175.3	0.795956	0.012053	0.997822
ENSSSCT00000081336	-1.46949	0.013311	0.997822

ENSSSCT00000089262	1.068713	0.013404	0.997822
ENSSSCT00000069708	-2.59856	0.013762	0.997822
ENSSSCT00000078212	6.876517	0.01393	0.997822
ENSSSCT00000087573	0.811459	0.015242	0.997822
MSTRG.14631.1	-1.41923	0.015574	0.997822
ENSSSCT00000084886	6.129283	0.016521	0.997822
ENSSSCT00000088022	-1.26303	0.016664	0.997822
MSTRG.5436.2	1.968587	0.016981	0.997822
MSTRG.13101.4	-1.60068	0.018134	0.997822
ENSSSCT00000073002	-3.90689	0.019581	0.997822
MSTRG.2510.1	0.656334	0.022227	0.997822
MSTRG.15295.3	-0.74651	0.022652	0.997822
ENSSSCT00000073304	-0.93089	0.02274	0.997822
MSTRG.941.1	-2.68517	0.023579	0.997822
ENSSSCT00000070825	-5.64386	0.024527	0.997822
MSTRG.16978.3	0.640116	0.024789	0.997822
ENSSSCT00000083033	1.584963	0.025478	0.997822
ENSSSCT00000073695	5.78136	0.02649	0.997822
MSTRG.5473.3	0.799557	0.026616	0.997822
MSTRG.2152.1	0.779553	0.027012	0.997822
MSTRG.327.5	0.698421	0.027034	0.997822
ENSSSCT00000087634	3	0.02734	0.997822
ENSSSCT00000076133	3.392317	0.028336	0.997822
MSTRG.7686.3	0.83243	0.029081	0.997822
MSTRG.13306.1	2.78061	0.030512	0.997822
ENSSSCT00000074339	2.169925	0.03066	0.997822
ENSSSCT00000077218	0.68226	0.031024	0.997822
ENSSSCT00000072673	1.547921	0.031467	0.997822
MSTRG.9353.1	0.751449	0.033397	0.997822

MSTRG.10504.3	2.19913	0.034551	0.997822
MSTRG.15695.1	0.649061	0.035135	0.997822
ENSSSCT00000077709	-5.24031	0.035248	0.997822
ENSSSCT00000069110	0.974689	0.03848	0.997822
MSTRG.10857.6	0.734914	0.039987	0.997822
ENSSSCT00000067704	-2.848	0.0416	0.997822
MSTRG.16419.1	-1.47573	0.041918	0.997822
MSTRG.3725.1	-1	0.042483	0.997822
ENSSSCT00000036447	-2.6231	0.043755	0.997822
ENSSSCT00000073277	0.910949	0.04458	0.997822
ENSSSCT00000068614	2.514573	0.045043	0.997822
ENSSSCT00000089982	-1.21791	0.045414	0.997822
ENSSSCT00000079896	-1.18057	0.046519	0.997822
ENSSSCT00000086943	1.302563	0.047603	0.997822
MSTRG.2194.1	1.466781	0.047718	0.997822
MSTRG.14918.6	1.409598	0.047747	0.997822
MSTRG.3133.4	-3.96963	0.048703	0.997822
ENSSSCT00000070586	3.584963	0.049524	0.997822
