# Supplementary material for: Identification and functional prediction of long non-coding RNAs related to skeletal muscle development in Duroc pigs
Source: Anim Biosci. 2022 Apr 30;35(10):1512–23. doi: 10.5713/ab.22.0020 (PMC9449383; doi:10.5713/ab.22.0020)
Supplement: Supplementary Table S9. — The list of DE mRNAs [file ab-22-0020-suppl9.pdf]

Table S9 The list of DE mRNAs

Sus scrofa Ensemble ID	log2(fc)	PValue	FDR
ENSSSCG00000015917	1.432136	1.01E-30	1.64E-26
ENSSSCG00000013155	0.692101	9.53E-14	7.78E-10
ENSSSCG00000011769	0.684276	3.75E-13	2.04E-09
ENSSSCG00000006752	0.676126	7.99E-13	3.26E-09
ENSSSCG00000033185	0.654188	1.99E-11	5.95E-08
ENSSSCG00000026098	0.859617	2.19E-11	5.95E-08
ENSSSCG00000003768	1.101058	9.13E-11	1.95E-07
ENSSSCG00000015310	0.705533	9.57E-11	1.95E-07
ENSSSCG00000029230	-0.75043	8.29E-10	1.50E-06
ENSSSCG00000013775	-0.71935	1.01E-09	1.64E-06
ENSSSCG00000004973	0.898263	1.59E-09	2.36E-06
ENSSSCG00000036083	0.850371	4.38E-09	5.52E-06
ENSSSCG00000004725	0.697226	4.40E-09	5.52E-06
ENSSSCG00000004691	0.844023	6.58E-09	7.67E-06
ENSSSCG00000005060	0.960056	8.12E-09	8.84E-06
ENSSSCG00000016640	0.683594	8.89E-09	9.07E-06
ENSSSCG00000029805	0.759613	1.48E-08	1.42E-05
ENSSSCG00000000457	0.852017	2.00E-08	1.81E-05
ENSSSCG00000013579	-1.34372	4.73E-08	3.68E-05
ENSSSCG00000014149	0.815456	6.72E-08	4.99E-05
ENSSSCG00000001075	0.780133	7.17E-08	5.09E-05
ENSSSCG00000005039	0.832923	8.54E-08	5.81E-05
ENSSSCG00000013344	0.645228	1.20E-07	7.83E-05
ENSSSCG00000010532	-1.61362	1.31E-07	8.22E-05
ENSSSCG00000006994	0.796837	2.39E-07	0.000131
ENSSSCG00000003105	-0.69675	2.41E-07	0.000131
ENSSSCG00000013402	0.613384	2.70E-07	0.000139

ENSSSCG00000005166	1.163958	3.48E-07	0.000167
ENSSSCG00000017024	0.59211	3.84E-07	0.000179
ENSSSCG00000027139	0.644822	4.34E-07	0.000197
ENSSSCG00000011027	0.633044	6.32E-07	0.000279
ENSSSCG00000004607	0.748379	7.51E-07	0.000323
ENSSSCG00000020702	0.87558	7.97E-07	0.000334
ENSSSCG00000021084	-0.84182	1.81E-06	0.000634
ENSSSCG00000033753	0.704176	1.82E-06	0.000634
ENSSSCG00000016916	0.643885	2.18E-06	0.00074
ENSSSCG00000032223	0.593864	2.41E-06	0.000802
ENSSSCG00000029198	0.653509	2.61E-06	0.000851
ENSSSCG00000027613	0.608178	3.56E-06	0.001139
ENSSSCG00000018086	0.726506	3.86E-06	0.001208
ENSSSCG00000035196	0.986642	5.10E-06	0.001488
ENSSSCG00000006137	0.671085	5.12E-06	0.001488
ENSSSCG00000003694	-0.58934	5.80E-06	0.001633
ENSSSCG00000035544	0.661318	6.09E-06	0.001685
ENSSSCG00000006395	-0.61802	7.01E-06	0.001907
ENSSSCG00000021217	0.613102	7.36E-06	0.001971
ENSSSCG00000010312	-1.04345	8.42E-06	0.002183
ENSSSCG00000035762	0.617261	1.20E-05	0.002878
ENSSSCG00000028025	0.58781	1.36E-05	0.003224
ENSSSCG00000027017	0.657156	1.52E-05	0.003549
ENSSSCG00000001770	-1.14156	1.59E-05	0.003609
ENSSSCG00000004652	0.608052	1.72E-05	0.003851
ENSSSCG00000015576	0.739305	1.96E-05	0.004327
ENSSSCG00000014924	-0.76381	2.62E-05	0.005619
ENSSSCG00000022236	-0.97302	3.03E-05	0.006188
ENSSSCG00000014316	-0.81785	3.20E-05	0.006444

ENSSSCG00000000536	0.596426	3.25E-05	0.006471
ENSSSCG00000015662	-0.94437	3.37E-05	0.006544
ENSSSCG00000037449	-0.69295	3.60E-05	0.006836
ENSSSCG00000011918	0.614755	3.78E-05	0.006976
ENSSSCG00000015446	0.744477	3.80E-05	0.006976
ENSSSCG00000028879	-0.62677	4.02E-05	0.007292
ENSSSCG00000049253	1.752072	4.30E-05	0.00772
ENSSSCG00000002368	-0.77407	4.47E-05	0.007918
ENSSSCG00000007522	-1.16244	4.51E-05	0.007918
ENSSSCG00000018091	0.773586	5.21E-05	0.008718
ENSSSCG00000001667	0.619455	5.23E-05	0.008718
ENSSSCG00000013297	-0.7658	5.75E-05	0.00938
ENSSSCG00000033608	-0.93432	5.81E-05	0.009392
ENSSSCG00000017472	-0.8191	6.58E-05	0.010124
ENSSSCG00000008017	-2.11662	6.64E-05	0.010124
ENSSSCG00000009668	-0.80377	6.70E-05	0.010124
ENSSSCG00000036491	0.898322	7.19E-05	0.010568
ENSSSCG00000017333	-0.84779	7.65E-05	0.010762
ENSSSCG00000007625	-0.78799	8.21E-05	0.011157
ENSSSCG00000003787	0.647223	8.34E-05	0.011157
ENSSSCG00000029371	-1.57905	8.47E-05	0.011236
ENSSSCG00000031261	0.873728	8.63E-05	0.011241
ENSSSCG00000008180	0.667887	8.64E-05	0.011241
ENSSSCG00000014321	0.620636	8.68E-05	0.011241
ENSSSCG00000036785	1.742414	9.70E-05	0.012067
ENSSSCG00000008096	0.659345	0.000111	0.013439
ENSSSCG00000026087	0.918946	0.000117	0.013763
ENSSSCG00000036742	0.589471	0.000119	0.013843
ENSSSCG00000002039	-0.67797	0.000125	0.014363

ENSSSCG00000001910	-0.70767	0.000143	0.015372
ENSSSCG00000018087	0.820117	0.000154	0.016033
ENSSSCG00000015840	-0.9969	0.000157	0.016089
ENSSSCG00000001427	-0.79075	0.000161	0.016413
ENSSSCG00000034139	0.658774	0.000163	0.01651
ENSSSCG00000028461	-0.92701	0.00018	0.017572
ENSSSCG00000031789	-0.70499	0.000182	0.017574
ENSSSCG00000028420	0.625105	0.000186	0.017815
ENSSSCG00000012652	-1.56786	0.000189	0.017816
ENSSSCG00000022536	-1.62192	0.000189	0.017816
ENSSSCG00000021166	1.031825	0.0002	0.018574
ENSSSCG00000010625	0.720346	0.000216	0.01956
ENSSSCG00000040056	0.670435	0.000223	0.020106
ENSSSCG00000022230	-0.5963	0.00023	0.020372
ENSSSCG00000015360	0.587295	0.000238	0.020914
ENSSSCG00000041081	1.180121	0.000241	0.021069
ENSSSCG00000010992	-0.80735	0.000274	0.023301
ENSSSCG00000018065	0.61785	0.000296	0.024518
ENSSSCG00000028113	0.616652	0.000304	0.025047
ENSSSCG00000040639	0.688056	0.000311	0.025354
ENSSSCG00000003231	-1.86426	0.000311	0.025354
ENSSSCG00000032193	-0.85792	0.000314	0.025354
ENSSSCG00000040731	0.63915	0.000315	0.025354
ENSSSCG00000001092	0.733147	0.000319	0.025524
ENSSSCG00000013895	-0.85449	0.000332	0.026092
ENSSSCG00000010433	0.608108	0.000356	0.027008
ENSSSCG00000012890	-0.69056	0.000404	0.029842
ENSSSCG00000004110	0.592722	0.000406	0.02986
ENSSSCG00000005271	0.691878	0.000409	0.029932

ENSSSCG00000001723	-0.84262	0.000413	0.030099
ENSSSCG00000036618	-1.27262	0.00043	0.031185
ENSSSCG00000037216	0.614278	0.000436	0.031513
ENSSSCG00000002860	0.849329	0.000448	0.031723
ENSSSCG00000032650	0.806764	0.000468	0.032542
ENSSSCG00000031788	-1.56768	0.000471	0.032611
ENSSSCG00000006610	-0.78024	0.000482	0.033121
ENSSSCG00000015559	-1.65436	0.000483	0.033121
ENSSSCG00000009061	0.624774	0.000509	0.034466
ENSSSCG00000017956	-1.43818	0.000516	0.034547
ENSSSCG00000008398	0.655311	0.000525	0.034844
ENSSSCG00000004634	0.621339	0.00056	0.036415
ENSSSCG00000008493	0.756331	0.000592	0.037917
ENSSSCG00000018069	0.855229	0.000598	0.038071
ENSSSCG00000002404	-0.74161	0.000608	0.038192
ENSSSCG00000015085	-1.32868	0.00064	0.03989
ENSSSCG00000031493	-0.92796	0.000656	0.040389
ENSSSCG00000040013	0.803052	0.000658	0.040397
ENSSSCG00000010606	0.645335	0.000666	0.040711
ENSSSCG00000011243	0.930614	0.000678	0.041043
ENSSSCG00000010600	-0.80828	0.000686	0.041043
ENSSSCG00000016298	-1.01735	0.000686	0.041043
ENSSSCG00000018078	0.594389	0.000694	0.041272
ENSSSCG00000015014	0.633172	0.000699	0.041272
ENSSSCG00000003700	0.691068	0.000703	0.041272
ENSSSCG00000037142	-0.79728	0.000728	0.042386
ENSSSCG00000035238	-0.61903	0.000735	0.042432
ENSSSCG00000012277	-1.12549	0.000736	0.042432
ENSSSCG00000028663	0.618366	0.000746	0.042573

ENSSSCG00000011106	-0.69315	0.000747	0.042573
ENSSSCG00000013876	-0.5857	0.000801	0.04479
ENSSSCG00000023297	0.657894	0.000817	0.045386
ENSSSCG00000024748	0.615435	0.000825	0.045496
ENSSSCG00000000142	-0.81453	0.000897	0.048018
ENSSSCG00000039408	-0.67731	0.000901	0.048084
ENSSSCG00000017498	-0.76961	0.000906	0.048186
ENSSSCG00000029608	0.69102	0.000978	0.051399
ENSSSCG00000033213	1.214912	0.001018	0.052768
ENSSSCG00000032591	-0.84841	0.001047	0.05391
ENSSSCG00000003458	-0.74846	0.001075	0.055206
ENSSSCG00000010648	0.813299	0.00112	0.056608
ENSSSCG00000016570	-0.97017	0.001125	0.056691
ENSSSCG00000009833	-0.68306	0.001164	0.057514
ENSSSCG00000007435	-1.26375	0.001171	0.057514
ENSSSCG00000006379	-1.68192	0.001173	0.057514
ENSSSCG00000036190	-9.77726	0.001239	0.059862
ENSSSCG00000003682	0.608295	0.001239	0.059862
ENSSSCG00000014010	0.627938	0.001258	0.060584
ENSSSCG00000016026	0.719892	0.001339	0.063398
ENSSSCG00000013731	-1.03758	0.00134	0.063398
ENSSSCG00000010184	-0.93517	0.0014	0.064724
ENSSSCG00000031712	-0.6664	0.00142	0.065099
ENSSSCG00000021585	-1.14886	0.001425	0.065139
ENSSSCG00000011398	-0.5853	0.001433	0.065147
ENSSSCG00000012844	-0.77672	0.001461	0.066044
ENSSSCG00000030603	2.075288	0.001483	0.066676
ENSSSCG00000014362	0.9265	0.001487	0.066676
ENSSSCG00000005984	0.589275	0.001508	0.067458

ENSSSCG00000011450	-1.58496	0.001526	0.06774
ENSSSCG00000013901	-1.18189	0.001589	0.069918
ENSSSCG00000008973	-1.46689	0.0016	0.070215
ENSSSCG00000002866	-0.67807	0.001638	0.071484
ENSSSCG00000037463	0.712863	0.001648	0.071753
ENSSSCG00000028814	-0.5908	0.001678	0.072845
ENSSSCG00000028905	0.686754	0.001718	0.073787
ENSSSCG00000021466	0.848441	0.001792	0.075934
ENSSSCG00000036246	-1.60112	0.001819	0.076547
ENSSSCG00000014840	-4.34577	0.001826	0.076645
ENSSSCG00000004983	0.698662	0.001855	0.07723
ENSSSCG00000012101	1.484436	0.001861	0.07723
MSTRG.16852	-4.13619	0.001864	0.07723
ENSSSCG00000006800	-1.197	0.001905	0.078503
ENSSSCG00000005494	-1.65219	0.00195	0.078903
ENSSSCG00000015938	0.657825	0.001953	0.078903
ENSSSCG00000010628	0.729412	0.001966	0.079153
ENSSSCG00000006506	-0.88204	0.001972	0.079153
ENSSSCG00000026960	0.629847	0.001973	0.079153
ENSSSCG00000012203	0.618814	0.002032	0.080829
ENSSSCG00000033849	-1.29442	0.002035	0.080829
ENSSSCG00000044815	6.569856	0.002177	0.084302
ENSSSCG00000039573	-1.49884	0.002179	0.084302
ENSSSCG00000008414	0.669386	0.002187	0.084417
ENSSSCG00000027487	-0.99487	0.002224	0.085414
ENSSSCG00000045502	-1.17834	0.002248	0.086126
ENSSSCG00000009931	0.62975	0.002268	0.086218
ENSSSCG00000034688	-1.17307	0.002269	0.086218
ENSSSCG00000013114	-1.17773	0.002282	0.086218

ENSSSCG00000036445	-8.00843	0.002284	0.086218
ENSSSCG00000038044	-0.72874	0.002298	0.086218
ENSSSCG00000012481	-1.1989	0.002356	0.087818
ENSSSCG00000031874	-0.72904	0.002394	0.088398
ENSSSCG00000037318	-0.70468	0.002405	0.088415
ENSSSCG00000012880	-1.03536	0.002458	0.089364
ENSSSCG00000004986	0.743508	0.002495	0.090125
ENSSSCG00000013766	-0.88716	0.002511	0.090475
ENSSSCG00000010672	0.585914	0.002525	0.090573
ENSSSCG00000033883	-0.61905	0.002573	0.091893
ENSSSCG00000016448	-0.67571	0.002583	0.091919
ENSSSCG00000037347	-8.12928	0.002607	0.092165
ENSSSCG00000003018	-0.78771	0.00264	0.092682
ENSSSCG00000017759	-0.63675	0.002668	0.092946
ENSSSCG00000017912	-0.65969	0.00267	0.092946
ENSSSCG00000017254	0.742001	0.002684	0.093107
ENSSSCG00000048408	1.027745	0.002686	0.093107
ENSSSCG00000036727	-1.1862	0.002692	0.093114
ENSSSCG00000030616	0.673054	0.002805	0.095623
ENSSSCG00000008937	-2.51706	0.002814	0.095643
ENSSSCG00000007436	-4.38082	0.002823	0.095643
ENSSSCG00000002009	-2.11548	0.002853	0.095712
ENSSSCG00000033512	-0.93099	0.002879	0.096122
ENSSSCG00000013313	-2.68806	0.002906	0.096621
ENSSSCG00000040095	-0.90689	0.002967	0.097985
ENSSSCG00000031616	0.629362	0.003007	0.098638
ENSSSCG00000014369	-1.1475	0.003034	0.098872
ENSSSCG00000045016	2.571542	0.003046	0.099039
ENSSSCG00000030660	0.633979	0.003095	0.100427

ENSSSCG00000016828	0.636231	0.003148	0.101554
ENSSSCG00000007151	-0.68816	0.00316	0.101751
ENSSSCG00000003451	-0.67157	0.003215	0.102298
ENSSSCG00000013377	-0.97366	0.003253	0.1033
ENSSSCG00000012556	0.714473	0.003266	0.103534
ENSSSCG00000011364	-0.84706	0.003314	0.104814
ENSSSCG00000000687	-0.8162	0.00332	0.104814
ENSSSCG00000013599	-1.05597	0.003451	0.107306
ENSSSCG00000006216	0.59173	0.003465	0.107539
ENSSSCG00000011436	-1.10692	0.003498	0.107933
ENSSSCG00000025955	-0.67807	0.003509	0.108004
ENSSSCG00000018081	0.626345	0.003533	0.108419
ENSSSCG00000013237	-1.01677	0.00371	0.111751
ENSSSCG00000005055	-0.86701	0.003737	0.112131
ENSSSCG00000017144	-1.7021	0.003768	0.112528
ENSSSCG00000017920	-1.04182	0.003771	0.112528
ENSSSCG00000000512	0.676145	0.003809	0.113255
ENSSSCG00000005446	-1.36923	0.003858	0.114301
ENSSSCG00000039056	-0.65844	0.00387	0.114455
ENSSSCG00000018080	1.230151	0.003892	0.114691
ENSSSCG00000011542	0.729241	0.003956	0.115295
ENSSSCG00000011640	-2.89724	0.003987	0.115295
ENSSSCG00000029811	0.641112	0.004009	0.115295
ENSSSCG00000020842	0.656006	0.00402	0.115295
ENSSSCG00000033411	0.585848	0.00404	0.115295
ENSSSCG00000015094	2.415037	0.004103	0.11679
ENSSSCG00000031118	-0.7885	0.004107	0.11679
ENSSSCG00000011916	-0.70712	0.004157	0.117816
ENSSSCG00000007574	-0.83667	0.004183	0.118133

ENSSSCG00000001422	-0.96253	0.004217	0.118897
ENSSSCG00000032086	0.655598	0.004239	0.118905
ENSSSCG00000000029	-0.77761	0.004268	0.119311
ENSSSCG00000038646	0.82875	0.004326	0.120262
ENSSSCG00000002917	-0.76458	0.00434	0.120293
ENSSSCG00000011304	1.082826	0.004462	0.122722
ENSSSCG00000024310	-0.9341	0.004481	0.122722
ENSSSCG00000037501	-1.15646	0.004492	0.122818
ENSSSCG00000001966	0.601173	0.00456	0.124092
ENSSSCG00000004446	0.587267	0.004563	0.124092
ENSSSCG00000008601	-1.04755	0.00457	0.124092
ENSSSCG00000001787	-0.70311	0.004596	0.124092
ENSSSCG00000010484	0.929345	0.004632	0.124561
ENSSSCG00000040445	0.653197	0.004641	0.124592
ENSSSCG00000008723	-0.6651	0.004674	0.12509
ENSSSCG00000028065	0.704684	0.004703	0.125583
ENSSSCG00000015709	0.608039	0.00477	0.126607
ENSSSCG00000004262	0.685267	0.004787	0.126677
ENSSSCG00000022208	-0.64438	0.004851	0.127709
ENSSSCG00000011610	-0.69916	0.004925	0.129043
ENSSSCG00000022190	0.618866	0.004966	0.129905
ENSSSCG00000013553	-1.10474	0.005017	0.131023
ENSSSCG00000000648	-7.49185	0.005096	0.132128
ENSSSCG00000011299	-0.63485	0.0051	0.132128
ENSSSCG00000021791	0.62922	0.005109	0.132128
ENSSSCG00000034570	-0.91102	0.005113	0.132128
ENSSSCG00000001132	-0.71635	0.005142	0.132184
ENSSSCG00000002452	-0.71321	0.005189	0.133011
ENSSSCG00000013400	-0.59764	0.005345	0.136119

ENSSSCG00000006495	-1.01017	0.005562	0.140342
ENSSSCG00000013888	-1.04103	0.0056	0.140637
ENSSSCG00000021944	-0.89681	0.005799	0.144533
ENSSSCG00000002919	-1.32225	0.005886	0.146047
ENSSSCG00000013498	-2.31564	0.005887	0.146047
ENSSSCG00000032428	-0.80792	0.005931	0.14674
ENSSSCG00000024437	-0.97818	0.005933	0.14674
ENSSSCG00000004779	-1.18763	0.005951	0.14674
ENSSSCG00000013892	-1.15161	0.006075	0.149113
ENSSSCG00000030300	-0.78549	0.006171	0.151253
ENSSSCG00000010688	-0.59054	0.006253	0.152625
ENSSSCG00000011510	0.592698	0.006328	0.154183
ENSSSCG00000021241	-1.52356	0.006385	0.155339
ENSSSCG00000013556	-1.68806	0.006495	0.1566
ENSSSCG00000023709	-0.78971	0.006534	0.15732
ENSSSCG00000003240	1.827819	0.006556	0.157544
ENSSSCG00000003137	-0.70531	0.006567	0.157544
ENSSSCG00000021436	-0.91723	0.006724	0.160713
ENSSSCG00000013604	-1.10737	0.006742	0.160912
ENSSSCG00000007797	-0.94886	0.006753	0.160935
ENSSSCG00000009880	-0.79317	0.006896	0.163625
ENSSSCG00000041777	0.760662	0.006909	0.163696
ENSSSCG00000013045	-10.0967	0.007065	0.166611
ENSSSCG00000036947	0.673003	0.007124	0.167568
ENSSSCG00000036152	-0.67982	0.007272	0.168758
ENSSSCG00000039745	-0.71555	0.007314	0.168758
ENSSSCG00000049794	4.273018	0.007472	0.171042
ENSSSCG00000026084	-0.7296	0.007685	0.174961
ENSSSCG00000014823	-1.11548	0.00787	0.178933

ENSSSCG00000002954	-1.38333	0.00797	0.180204
ENSSSCG00000007366	-1.39855	0.00804	0.181035
ENSSSCG00000002037	-1.41504	0.008217	0.183219
ENSSSCG00000008614	0.650551	0.008226	0.183219
ENSSSCG00000006400	-6.49185	0.00826	0.183697
ENSSSCG00000035170	-1.13905	0.008315	0.183955
ENSSSCG00000010683	-0.74328	0.008318	0.183955
ENSSSCG00000017306	-0.65921	0.008381	0.184536
ENSSSCG00000009625	-2.09818	0.008399	0.184536
ENSSSCG00000025034	-1.28577	0.008441	0.184876
ENSSSCG00000040798	-1.22465	0.008449	0.184876
ENSSSCG00000011322	-1.41954	0.008545	0.186235
ENSSSCG00000006811	-0.875	0.008643	0.187854
ENSSSCG00000012397	-1.48175	0.008764	0.189485
ENSSSCG00000000779	0.606464	0.008839	0.189847
ENSSSCG00000007003	-0.86143	0.00893	0.191047
ENSSSCG00000034572	1.009586	0.009	0.1923
ENSSSCG00000020884	-3.90689	0.009099	0.193917
ENSSSCG00000016751	0.758325	0.009164	0.195039
ENSSSCG00000028201	0.703421	0.009431	0.198884
ENSSSCG00000025685	-1.3505	0.009454	0.198884
ENSSSCG00000038804	1.936806	0.009587	0.200638
ENSSSCG00000032637	-0.61771	0.009661	0.201268
ENSSSCG00000016851	-0.69793	0.009672	0.201268
ENSSSCG00000041431	2.906891	0.009744	0.201351
ENSSSCG00000027516	0.851298	0.009757	0.201351
ENSSSCG00000009396	-0.63152	0.009816	0.201816
ENSSSCG00000027124	-0.84719	0.009831	0.201871
ENSSSCG00000017087	-0.87882	0.00992	0.202927

ENSSSCG00000008834	3.807355	0.010031	0.204537
ENSSSCG00000002035	-0.95161	0.010369	0.209371
ENSSSCG00000035908	-1.53605	0.010404	0.209676
ENSSSCG00000000734	-0.69338	0.010501	0.211371
ENSSSCG00000010450	-0.83598	0.010524	0.211575
ENSSSCG00000038706	-0.76858	0.010555	0.211874
ENSSSCG00000002960	-0.97369	0.010565	0.211874
ENSSSCG00000000647	-2.22517	0.010636	0.212768
ENSSSCG00000023771	-4.44294	0.01068	0.213137
ENSSSCG00000003558	-0.85971	0.010757	0.214404
ENSSSCG00000048968	4.523562	0.010802	0.214721
ENSSSCG00000024024	-2.05063	0.010866	0.214785
ENSSSCG00000040252	0.619396	0.01091	0.215088
ENSSSCG00000038600	-1.69642	0.010967	0.215436
ENSSSCG00000036747	-1.03892	0.010984	0.215454
ENSSSCG00000037567	0.610241	0.010994	0.215454
ENSSSCG00000031498	0.736966	0.011024	0.215646
ENSSSCG00000050480	2.364572	0.011031	0.215646
ENSSSCG00000037241	-0.93427	0.011066	0.215816
ENSSSCG00000033451	-1.00174	0.011138	0.216309
ENSSSCG00000045005	2.490326	0.011203	0.21707
ENSSSCG00000017407	-1.2202	0.011322	0.218721
ENSSSCG00000033355	-2.03747	0.011382	0.219592
ENSSSCG00000003975	-0.70634	0.011419	0.219818
ENSSSCG00000002287	0.6822	0.01147	0.220285
ENSSSCG00000027196	-1.16358	0.01165	0.2232
ENSSSCG00000016497	-0.87023	0.011664	0.223215
ENSSSCG00000014843	-1.44283	0.011697	0.223577
ENSSSCG00000017608	-0.63919	0.011867	0.226313

ENSSSCG00000011443	-0.68737	0.011958	0.227133
ENSSSCG00000017733	0.725825	0.011962	0.227133
ENSSSCG00000008069	2.06301	0.012058	0.228085
ENSSSCG00000005453	-0.59162	0.012119	0.228706
ENSSSCG00000017235	-1.73697	0.012158	0.228871
ENSSSCG00000013517	-1.51896	0.01217	0.228871
ENSSSCG00000015577	0.588629	0.012334	0.230816
ENSSSCG00000040419	0.770518	0.01235	0.230816
ENSSSCG00000034284	-1.0125	0.012587	0.23323
ENSSSCG00000006546	-1.06515	0.01262	0.233339
ENSSSCG00000026943	-1.21853	0.012713	0.234223
ENSSSCG00000039874	-1	0.01276	0.234824
ENSSSCG00000003088	-0.64794	0.012797	0.235102
ENSSSCG00000008239	-0.90361	0.012804	0.235102
ENSSSCG00000038477	1.5025	0.012918	0.236671
ENSSSCG00000013393	-0.82021	0.013191	0.239256
ENSSSCG00000014244	0.644945	0.013206	0.239256
ENSSSCG00000013853	-0.84528	0.013295	0.240204
ENSSSCG00000016573	-0.83776	0.013303	0.240204
ENSSSCG00000014899	-0.71436	0.013556	0.242835
MSTRG.12025	-0.96242	0.013608	0.242835
ENSSSCG00000002620	-0.6511	0.013627	0.242835
ENSSSCG00000025795	-1.40054	0.013698	0.243102
ENSSSCG00000035218	-1.62323	0.013971	0.246291
ENSSSCG00000006184	0.718336	0.014205	0.249877
ENSSSCG00000003524	-0.78371	0.014236	0.250142
ENSSSCG00000009197	6.609794	0.014255	0.25021
ENSSSCG00000024428	-1.8024	0.014316	0.25101
ENSSSCG00000008948	-4.8009	0.01435	0.251336

ENSSSCG00000000130	-0.93914	0.014478	0.25277
ENSSSCG00000016008	0.774933	0.014578	0.253776
ENSSSCG00000017343	-1.17232	0.01458	0.253776
ENSSSCG00000006309	-0.60577	0.014697	0.255231
ENSSSCG00000006218	0.722509	0.014832	0.256214
ENSSSCG00000006372	-0.83906	0.014885	0.256851
ENSSSCG00000013100	-0.93996	0.014917	0.256966
ENSSSCG00000020711	0.777608	0.014967	0.257428
ENSSSCG00000016957	-3.06413	0.015027	0.257814
ENSSSCG00000012172	-0.73256	0.015035	0.257814
ENSSSCG00000028840	-1.1244	0.015074	0.258206
MSTRG.17003	0.765743	0.01519	0.258587
ENSSSCG00000033030	0.661655	0.0152	0.258587
ENSSSCG00000006979	-2.26053	0.015223	0.258587
ENSSSCG00000007240	-1.64103	0.015295	0.258998
ENSSSCG00000039392	1.099536	0.015361	0.259449
ENSSSCG00000028523	0.68126	0.01537	0.259449
ENSSSCG00000010772	-1.34927	0.015439	0.259449
ENSSSCG00000017258	-0.91191	0.015532	0.259507
ENSSSCG00000017991	-2.15071	0.015579	0.260035
ENSSSCG00000036961	-3.11974	0.0156	0.260105
ENSSSCG00000025912	0.598259	0.015615	0.260105
ENSSSCG00000035243	1.584963	0.015665	0.260138
ENSSSCG00000025133	-1.34384	0.015696	0.260382
ENSSSCG00000009341	0.663475	0.016051	0.264235
ENSSSCG00000017376	-0.70722	0.016066	0.264235
ENSSSCG00000029005	-0.75979	0.01609	0.264235
ENSSSCG00000033674	-1.11042	0.016117	0.264235
ENSSSCG00000031970	-1.03413	0.016122	0.264235

ENSSSCG00000040981	-1.60567	0.016396	0.268187
ENSSSCG00000012492	-0.79189	0.016487	0.26914
ENSSSCG00000047166	2.032421	0.016551	0.269654
ENSSSCG00000003219	2.584963	0.01671	0.270633
MSTRG.10504	1.637192	0.016711	0.270633
ENSSSCG00000012053	-1.16816	0.016785	0.270995
ENSSSCG00000006288	-1.10309	0.0168	0.270995
ENSSSCG00000004033	-0.73989	0.016932	0.27203
ENSSSCG00000035442	-0.71319	0.016948	0.27203
ENSSSCG00000006312	0.797745	0.017035	0.272895
ENSSSCG00000004736	-2.32193	0.017127	0.274031
ENSSSCG00000006578	-1.11045	0.017316	0.274708
ENSSSCG00000036056	-0.97199	0.017367	0.275239
ENSSSCG00000016164	0.872426	0.017401	0.27551
ENSSSCG00000037257	-5.90689	0.017589	0.277147
ENSSSCG00000030069	-0.93887	0.017636	0.277622
ENSSSCG00000030371	-7.32193	0.017692	0.277872
ENSSSCG00000045684	0.922595	0.017721	0.277872
ENSSSCG00000014132	0.732896	0.017737	0.277872
ENSSSCG00000025588	-0.91278	0.01785	0.27883
ENSSSCG00000038158	0.682938	0.018024	0.280747
ENSSSCG00000005503	-0.848	0.018212	0.281847
ENSSSCG00000017062	-2.47805	0.01826	0.282008
ENSSSCG00000032698	-0.71109	0.018364	0.283337
ENSSSCG00000002980	-3.31792	0.018418	0.283776
ENSSSCG00000004710	0.675092	0.018567	0.284862
ENSSSCG00000040824	-3.80735	0.018632	0.285583
ENSSSCG00000004065	0.647823	0.018801	0.28631
ENSSSCG00000026947	-0.61631	0.01882	0.28631

ENSSSCG00000017918	-0.65208	0.018921	0.286851
ENSSSCG00000024692	-0.60402	0.018925	0.286851
ENSSSCG00000016452	-1.70044	0.019019	0.287922
ENSSSCG00000035697	3.5025	0.019434	0.291581
ENSSSCG00000033457	-1.27684	0.019573	0.292441
ENSSSCG00000027826	-1.62043	0.019581	0.292441
ENSSSCG00000036011	-1.25432	0.019678	0.293629
ENSSSCG00000026890	-1.88452	0.019727	0.293712
ENSSSCG00000031121	0.91427	0.019907	0.295417
ENSSSCG00000005240	-0.80735	0.019984	0.296195
ENSSSCG00000023908	0.863938	0.020041	0.296593
ENSSSCG00000015290	-0.8256	0.020092	0.29681
ENSSSCG00000009517	-2	0.020132	0.297133
ENSSSCG00000026653	-1.53267	0.02047	0.300093
ENSSSCG00000009818	0.805497	0.020585	0.300737
ENSSSCG00000016579	-1.20353	0.020597	0.300737
ENSSSCG00000021161	-1.12902	0.020618	0.300782
ENSSSCG00000008147	-0.5967	0.020911	0.303617
ENSSSCG00000022478	-0.61229	0.020962	0.303617
ENSSSCG00000025992	-3.32193	0.021095	0.304669
ENSSSCG00000036454	0.749707	0.021138	0.304818
ENSSSCG00000027565	-0.85157	0.021414	0.308156
ENSSSCG00000013352	-3.61471	0.021426	0.308156
ENSSSCG00000000171	-0.65192	0.021565	0.309137
ENSSSCG00000003949	-1.4021	0.02157	0.309137
ENSSSCG00000014274	-0.5946	0.021638	0.309848
ENSSSCG00000003170	3.906891	0.021784	0.311339
ENSSSCG00000026852	-1.29574	0.021829	0.311339
ENSSSCG00000029815	-0.65896	0.022037	0.313359

ENSSSCG00000043411	1.071791	0.022158	0.314455
ENSSSCG00000015802	-1.07982	0.022439	0.315471
ENSSSCG00000050591	6.179909	0.02255	0.316242
ENSSSCG00000025116	-0.63119	0.022623	0.316242
ENSSSCG00000006648	-0.89355	0.022627	0.316242
ENSSSCG00000006187	-0.88504	0.022829	0.317975
ENSSSCG00000033912	0.812914	0.023035	0.318913
ENSSSCG00000000050	-0.60878	0.023053	0.318913
ENSSSCG00000023996	0.630243	0.023214	0.32033
ENSSSCG00000010077	-3.53605	0.023247	0.320418
ENSSSCG00000032019	-1.34975	0.023324	0.320763
ENSSSCG00000012909	-0.7727	0.023458	0.321864
ENSSSCG00000037682	-0.81209	0.023481	0.321864
ENSSSCG00000016052	-0.83289	0.023893	0.32608
ENSSSCG00000012077	-0.85083	0.024067	0.327696
ENSSSCG00000039270	-2.45337	0.024125	0.327901
ENSSSCG00000051205	-1.09039	0.024224	0.328438
ENSSSCG00000002781	3.087463	0.024255	0.328581
ENSSSCG00000007146	-1.18982	0.024354	0.329481
ENSSSCG00000045169	-3.16993	0.024382	0.329481
ENSSSCG00000033106	2.042644	0.024445	0.330052
ENSSSCG00000009373	-0.71348	0.0245	0.33053
ENSSSCG00000045622	-2.73697	0.024555	0.330992
MSTRG.16850	-4.08342	0.024847	0.333679
ENSSSCG00000000206	-0.64508	0.024959	0.334511
ENSSSCG00000024972	0.64174	0.024981	0.334533
ENSSSCG00000013576	-1.38187	0.025056	0.334978
ENSSSCG00000013880	-4.64386	0.025105	0.335239
ENSSSCG00000039770	-0.89383	0.025238	0.336313

ENSSSCG00000004053	-1.04002	0.025427	0.33701
ENSSSCG00000004789	-0.6885	0.025501	0.337347
ENSSSCG00000002651	-1.53952	0.025571	0.337987
ENSSSCG00000047815	5.643856	0.025836	0.340375
ENSSSCG00000016475	-1.02466	0.025836	0.340375
ENSSSCG00000023630	-1.16721	0.025875	0.340375
ENSSSCG00000011915	1.708951	0.02588	0.340375
ENSSSCG00000016859	-1.30378	0.025897	0.340375
ENSSSCG00000011700	2.137504	0.025942	0.340692
ENSSSCG00000001773	-0.7219	0.026034	0.341554
ENSSSCG00000044858	2.765535	0.02605	0.341554
ENSSSCG00000033514	0.631699	0.0266	0.346724
ENSSSCG00000036469	-0.82996	0.026921	0.348498
ENSSSCG00000050253	3.459432	0.027121	0.349929
ENSSSCG00000021748	-0.86657	0.027402	0.351329
ENSSSCG00000041149	2.887525	0.027423	0.351329
ENSSSCG00000046587	-1.79751	0.027615	0.351944
ENSSSCG00000016705	-1.04385	0.027621	0.351944
ENSSSCG00000034211	0.752562	0.027661	0.351944
ENSSSCG00000005090	0.770749	0.028072	0.354208
ENSSSCG00000007323	0.838081	0.028263	0.354902
ENSSSCG00000027179	2.510962	0.028383	0.355858
ENSSSCG00000016943	0.619256	0.028688	0.358858
ENSSSCG00000040208	-0.77887	0.02882	0.359948
ENSSSCG00000038345	-0.76877	0.029007	0.360808
ENSSSCG00000014670	-0.7688	0.029027	0.360808
ENSSSCG00000021757	1.129283	0.029212	0.360808
ENSSSCG00000009182	-9.51175	0.029234	0.360808
ENSSSCG00000035760	1.222392	0.029276	0.360808

ENSSSCG00000024685	-3.04439	0.029335	0.360808
ENSSSCG00000009475	-2.14684	0.029366	0.360808
ENSSSCG00000035256	-0.69599	0.029368	0.360808
ENSSSCG00000050715	-2.85798	0.029375	0.360808
ENSSSCG00000028108	-0.62476	0.029397	0.360815
ENSSSCG00000000295	-0.94919	0.029575	0.362172
ENSSSCG00000013664	-0.76344	0.029784	0.363919
ENSSSCG00000039660	-6.02237	0.029904	0.364477
ENSSSCG00000013042	-1.20945	0.029955	0.364477
ENSSSCG00000017008	-0.84246	0.030328	0.367488
ENSSSCG00000000257	-0.9771	0.030378	0.367488
ENSSSCG00000014441	-0.94657	0.030391	0.367488
ENSSSCG00000030002	0.735297	0.030547	0.368641
ENSSSCG00000036352	-1.7559	0.030662	0.36877
ENSSSCG00000015742	0.692956	0.030861	0.369907
ENSSSCG00000011723	0.845025	0.030863	0.369907
ENSSSCG00000047351	5.965784	0.031041	0.371495
ENSSSCG00000009460	0.591596	0.03137	0.373078
ENSSSCG00000007754	-1.38833	0.031502	0.373078
ENSSSCG00000009747	0.796908	0.031504	0.373078
ENSSSCG00000011358	-0.79463	0.031529	0.373078
ENSSSCG00000031640	-0.61785	0.031561	0.373078
ENSSSCG00000029943	1.34915	0.03161	0.373078
ENSSSCG00000010639	0.635946	0.031626	0.373078
ENSSSCG00000021464	-0.79727	0.031654	0.373078
ENSSSCG00000028204	-3.86129	0.031731	0.373627
ENSSSCG00000033235	-1.75207	0.031811	0.373825
ENSSSCG00000009002	-1.2863	0.031848	0.373825
ENSSSCG00000011186	1.137504	0.031919	0.374311

ENSSSCG00000006100	1.217818	0.032435	0.378738
ENSSSCG00000000136	-0.93377	0.032469	0.378854
ENSSSCG00000024312	-1.37638	0.032587	0.379778
ENSSSCG00000025770	-0.74416	0.032594	0.379778
ENSSSCG00000033025	1.206451	0.032686	0.379928
ENSSSCG00000006287	-1.02405	0.032694	0.379928
ENSSSCG00000032609	-1.17776	0.032715	0.379928
ENSSSCG00000024152	-0.86889	0.032777	0.380003
ENSSSCG00000038540	-0.76867	0.033013	0.381412
ENSSSCG00000039823	-2.63227	0.033332	0.381854
ENSSSCG00000002292	-1.21459	0.033355	0.381854
ENSSSCG00000006734	-1.82312	0.03357	0.382302
ENSSSCG00000022004	-0.58551	0.033813	0.383307
ENSSSCG00000039926	-1.04901	0.034037	0.385309
ENSSSCG00000051213	-3.16993	0.034202	0.386018
ENSSSCG00000006569	-0.8719	0.0343	0.386679
ENSSSCG00000008311	-1.31228	0.034519	0.388618
ENSSSCG00000023865	1.441802	0.034689	0.389453
ENSSSCG00000026742	-1.3388	0.034859	0.390289
ENSSSCG00000028637	3.459432	0.035028	0.391915
ENSSSCG00000026850	-1.05445	0.035159	0.392377
ENSSSCG00000033222	-0.70823	0.035199	0.392482
ENSSSCG00000037918	-0.68932	0.035258	0.392599
ENSSSCG00000025704	-1.25239	0.035499	0.394024
ENSSSCG00000009753	-3.16993	0.035548	0.394081
ENSSSCG00000032623	-1.01336	0.035584	0.394081
ENSSSCG00000023933	-2.20163	0.035791	0.395569
ENSSSCG00000017705	-1.17066	0.0359	0.395837
ENSSSCG00000015781	-1.39102	0.035904	0.395837

ENSSSCG00000033721	-0.88982	0.035904	0.395837
ENSSSCG00000032198	0.622362	0.035977	0.396014
ENSSSCG00000033909	-1.03349	0.036019	0.396211
MSTRG.15673	0.820831	0.036264	0.396991
ENSSSCG00000007568	-0.81791	0.036308	0.396991
ENSSSCG00000027594	0.62849	0.036309	0.396991
ENSSSCG00000007944	-0.65426	0.036734	0.399615
ENSSSCG00000039798	-1.53605	0.036804	0.399615
ENSSSCG00000005135	-0.75576	0.036961	0.400661
ENSSSCG00000038851	-3.32193	0.037005	0.400715
ENSSSCG00000000660	-2.14852	0.037042	0.400715
ENSSSCG00000031700	-5.56986	0.037363	0.40192
ENSSSCG00000028536	-0.60192	0.03761	0.402854
ENSSSCG00000027144	2.459432	0.037636	0.402854
ENSSSCG00000003981	0.714843	0.037648	0.402854
ENSSSCG00000032355	-1.71969	0.037746	0.402854
ENSSSCG00000022773	-0.74322	0.037801	0.402854
ENSSSCG00000027628	-0.59869	0.037836	0.402893
ENSSSCG00000035863	-0.6712	0.038089	0.404674
ENSSSCG00000002275	-2	0.038102	0.404674
ENSSSCG00000032392	1.012824	0.038145	0.404866
ENSSSCG00000039416	-0.59107	0.038272	0.405679
ENSSSCG00000015125	-0.79113	0.03832	0.405926
ENSSSCG00000008984	-1.87915	0.038591	0.407602
MSTRG.11293	-1.7879	0.038715	0.407927
ENSSSCG00000028479	-1.691	0.038759	0.407927
ENSSSCG00000010084	-1.76429	0.039007	0.409222
ENSSSCG00000040486	0.668486	0.039095	0.409882
ENSSSCG00000015961	-1.77761	0.03918	0.410515

ENSSSCG00000007739	-0.59883	0.03956	0.413163
ENSSSCG00000037481	0.780069	0.039604	0.413363
ENSSSCG00000013989	-1.361	0.039706	0.413897
ENSSSCG00000030197	0.614019	0.040027	0.415592
ENSSSCG00000028345	0.760049	0.040047	0.415592
ENSSSCG00000033880	-1.93289	0.040194	0.416427
ENSSSCG00000006799	0.790077	0.040265	0.416535
MSTRG.12227	1.249778	0.040293	0.416556
ENSSSCG00000022483	-3.72247	0.040389	0.417026
ENSSSCG00000029414	-1.22764	0.040526	0.417225
ENSSSCG00000016504	-1.28226	0.040537	0.417225
ENSSSCG00000025644	-2.0319	0.040881	0.419234
ENSSSCG00000011344	0.873965	0.041227	0.421934
ENSSSCG00000013331	0.677179	0.041944	0.424744
ENSSSCG00000027415	-1.02137	0.042074	0.425144
ENSSSCG00000024622	0.710493	0.042107	0.425144
ENSSSCG00000002494	-0.79189	0.042114	0.425144
ENSSSCG00000023743	0.911773	0.042131	0.425144
ENSSSCG00000042270	-4.04439	0.042169	0.425144
ENSSSCG00000039442	0.66684	0.042179	0.425144
ENSSSCG00000006357	-0.73043	0.042202	0.425144
ENSSSCG00000024419	0.754888	0.042394	0.425144
ENSSSCG00000040047	-1.10962	0.042941	0.427943
ENSSSCG00000022492	-1.09982	0.043031	0.428576
ENSSSCG00000006329	-1.54057	0.04309	0.428902
ENSSSCG00000037491	-1.6911	0.043243	0.429728
ENSSSCG00000014275	-2.20945	0.043303	0.429728
ENSSSCG00000042040	1.584963	0.043316	0.429728
ENSSSCG00000038055	-0.65428	0.043323	0.429728

ENSSSCG00000032950	-0.62052	0.043823	0.432509
ENSSSCG00000015756	-5.71425	0.044234	0.433505
ENSSSCG00000003573	-0.62071	0.044243	0.433505
ENSSSCG00000032167	-3.5025	0.044426	0.43426
ENSSSCG00000024681	0.839064	0.044549	0.434769
ENSSSCG00000007524	1.047306	0.044611	0.435028
ENSSSCG00000003601	-1.91754	0.044933	0.436171
ENSSSCG00000002033	-3.48543	0.045067	0.436779
ENSSSCG00000050558	3.584963	0.04517	0.436779
ENSSSCG00000005510	-0.59589	0.045234	0.436779
ENSSSCG00000033146	-1.06266	0.045438	0.437343
ENSSSCG00000016686	-3.49185	0.045511	0.437523
ENSSSCG00000023160	-0.72357	0.04563	0.438152
ENSSSCG00000007788	-0.6223	0.045745	0.438746
ENSSSCG00000004531	0.713119	0.045784	0.438863
ENSSSCG00000038677	-3.10434	0.045863	0.439062
ENSSSCG00000001561	-1.61005	0.045947	0.439063
ENSSSCG00000002650	-0.66811	0.046213	0.440659
ENSSSCG00000009412	-1.24722	0.046294	0.440928
ENSSSCG00000010509	-0.89308	0.046312	0.440928
ENSSSCG00000016657	-5.02237	0.046464	0.442001
ENSSSCG00000012006	-1.23447	0.046563	0.44259
ENSSSCG00000012074	-0.6755	0.046688	0.44259
ENSSSCG00000023298	-0.98118	0.046968	0.444213
ENSSSCG00000016782	-0.89642	0.047338	0.445899
ENSSSCG00000033581	1.70044	0.047569	0.447177
ENSSSCG00000042694	2.584963	0.047693	0.447693
ENSSSCG00000032145	0.830942	0.047724	0.447726
ENSSSCG00000031620	3.681824	0.047921	0.4488

ENSSSCG00000027571	0.835464	0.047978	0.44908
ENSSSCG00000035592	-3.24793	0.048183	0.449983
ENSSSCG00000003414	-1.71823	0.048213	0.449985
ENSSSCG00000013106	-0.6622	0.048523	0.45108
ENSSSCG00000050966	0.851901	0.049046	0.452627
ENSSSCG00000033453	-0.65102	0.04905	0.452627
ENSSSCG00000002028	0.729873	0.049374	0.454627
ENSSSCG00000031450	-1.22211	0.049453	0.454627
ENSSSCG00000012548	0.655241	0.04976	0.455575
