# Supplementary material for: Identification and functional prediction of long non-coding RNAs related to skeletal muscle development in Duroc pigs
Source: Anim Biosci. 2022 Apr 30;35(10):1512–23. doi: 10.5713/ab.22.0020 (PMC9449383; doi:10.5713/ab.22.0020)
Supplement: Supplementary Table S10. — Analysis of traits [file ab-22-0020-suppl10.pdf]

Table S10 Analysis of traits

Traits	H group	L group
30-110 kg body weight ADG	768.75±12.34	758.25±17.97

Note: [a, b] Those with the different letters in each line showed significant difference (*P*<0.05). Those with no letters in each line showed no significant difference(*P*>0.05).
