# Supplementary material for: Identification and functional prediction of long non-coding RNAs related to skeletal muscle development in Duroc pigs
Source: Anim Biosci. 2022 Apr 30;35(10):1512–23. doi: 10.5713/ab.22.0020 (PMC9449383; doi:10.5713/ab.22.0020)
Supplement: Supplementary Figure S1. — Statistics of Transcription factors (TFs). The abscissa is the TF family, the ordinate is the number of genes. [file ab-22-0020-suppl11.pdf]

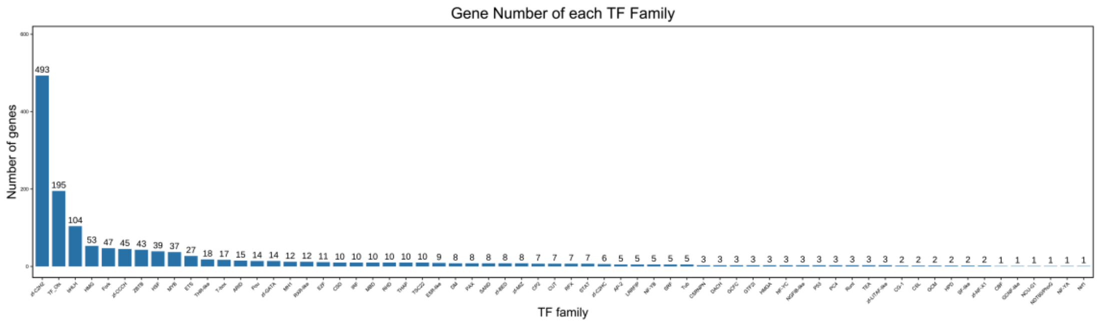

Figure S1. Statistics of Transcription factors (TFs). The abscissa is the TF family, the ordinate is the number of genes.
