# Supplementary material for: Identification and functional prediction of long non-coding RNAs related to skeletal muscle development in Duroc pigs
Source: Anim Biosci. 2022 Apr 30;35(10):1512–23. doi: 10.5713/ab.22.0020 (PMC9449383; doi:10.5713/ab.22.0020)
Supplement: Supplementary Figure S2. — Analysis of lncRNA type and the chromosome distribution of identified lncRNAs. [file ab-22-0020-suppl12.pdf]

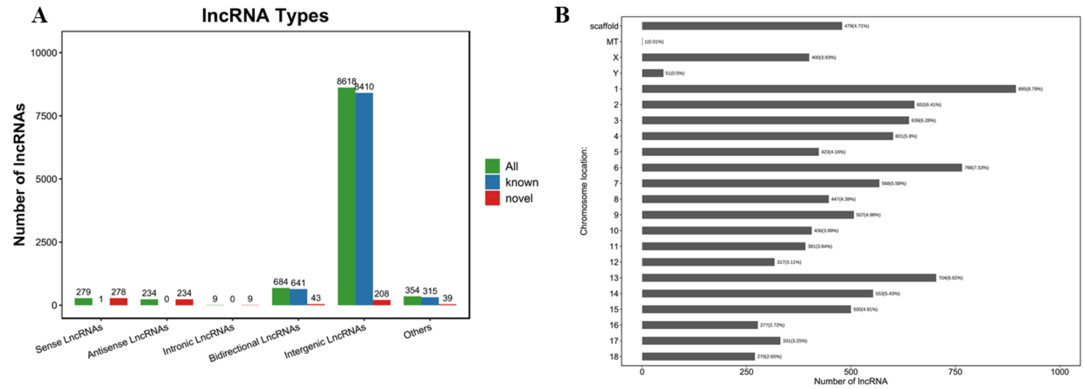

Figure S2. Analysis of lncRNA type and the chromosome distribution of identified lncRNAs. **A:** Analysis of lncRNA type. The abscissa represents lncRNA Type, the ordinate is the number of lncRNAs, **B:** The chromosome distribution of identified lncRNAs. The abscissa represents the number of lncRNAs, the ordinate represents Chromosome location.
