# Supplementary material for: Identification and functional prediction of long non-coding RNAs related to skeletal muscle development in Duroc pigs
Source: Anim Biosci. 2022 Apr 30;35(10):1512–23. doi: 10.5713/ab.22.0020 (PMC9449383; doi:10.5713/ab.22.0020)
Supplement: Supplementary Figure S3. — Comparison of genomic characteristics between lncRNAs and mRNAs. [file ab-22-0020-suppl13.pdf]

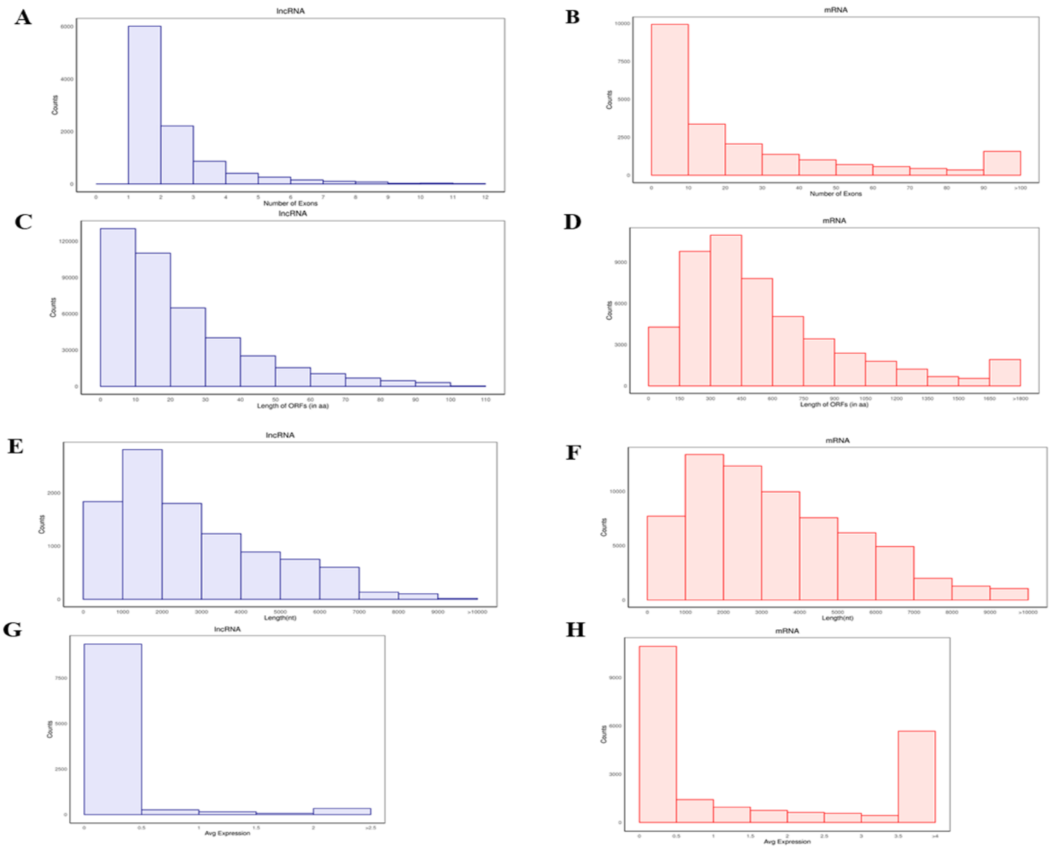

Figure S3. Comparison of genomic characteristics between lncRNAs and mRNAs. **A, B:** Number of lncRNAs and mRNAs exons. **C, D:** Length of lncRNAs and mRNAs ORF. **E, F:** Transcript length of lncRNAs and mRNAs. **G, H:** The expression levels of lncRNAs and mRNAs.
