# Supplementary material for: Identification and functional prediction of long non-coding RNAs related to skeletal muscle development in Duroc pigs
Source: Anim Biosci. 2022 Apr 30;35(10):1512–23. doi: 10.5713/ab.22.0020 (PMC9449383; doi:10.5713/ab.22.0020)
Supplement: Supplementary Figure S4. — The first 20 GO terms. [file ab-22-0020-suppl14.pdf]

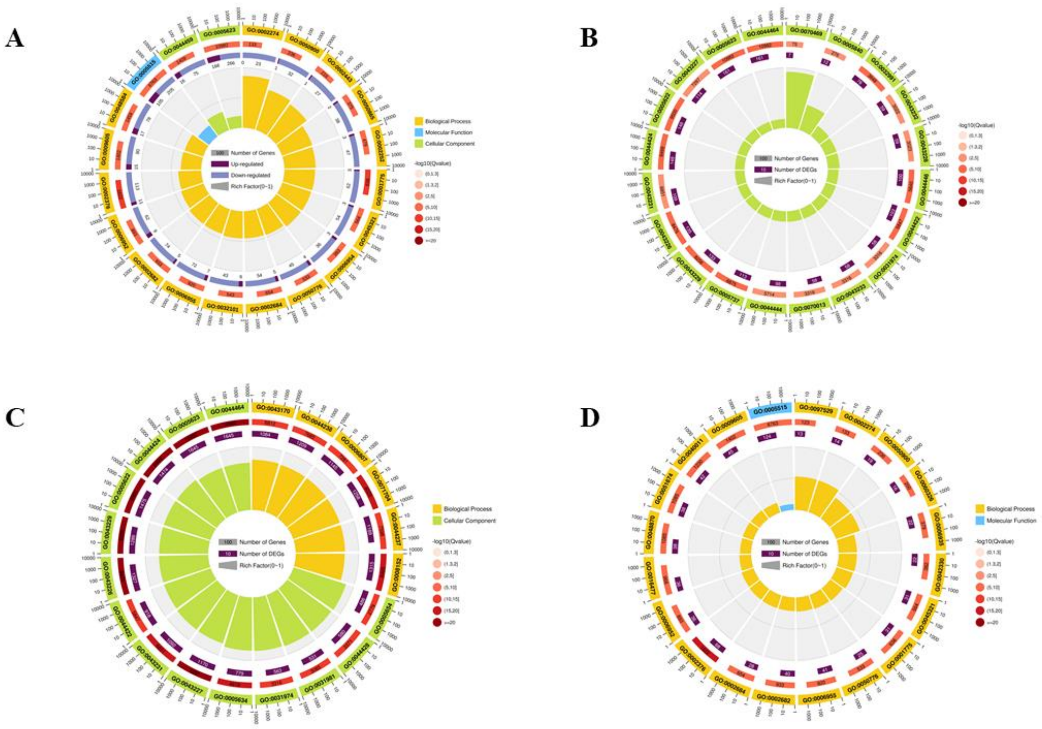

Figure S4. The first 20 GO terms. **A:** DEGs. **B:** Target genes of antisense regulation lncRNAs. **C:** Target genes of cis regulation lncRNAs. **D:** Target genes of trans regulation lncRNAs. The first circle: the top 20 GO terms were enriched, outside the circle is the coordinate ruler of the number of differential genes. Different colors represent different Ontology; the second circle: the number and Q value of the GO term in the DE gene background. The greater the number of DE genes background, the longer the bar; the smaller the Q value, the redder the color; the third circle: the bar graph of the ratio of up-regulated and down-regulated DE genes, dark purple represents the ratio of up-regulated DE genes, and light purple represents the ratio of down-regulated DE genes; the lower part shows the specific value; the fourth circle: the RichFactor value of each GO term.
