# Supplementary material for: Identification and functional prediction of long non-coding RNAs related to skeletal muscle development in Duroc pigs
Source: Anim Biosci. 2022 Apr 30;35(10):1512–23. doi: 10.5713/ab.22.0020 (PMC9449383; doi:10.5713/ab.22.0020)
Supplement: Supplementary Figure S5. — Electropherogram analysis of Total RNA on Agilent 2100 Bioanalyzer. [file ab-22-0020-suppl15.pdf]

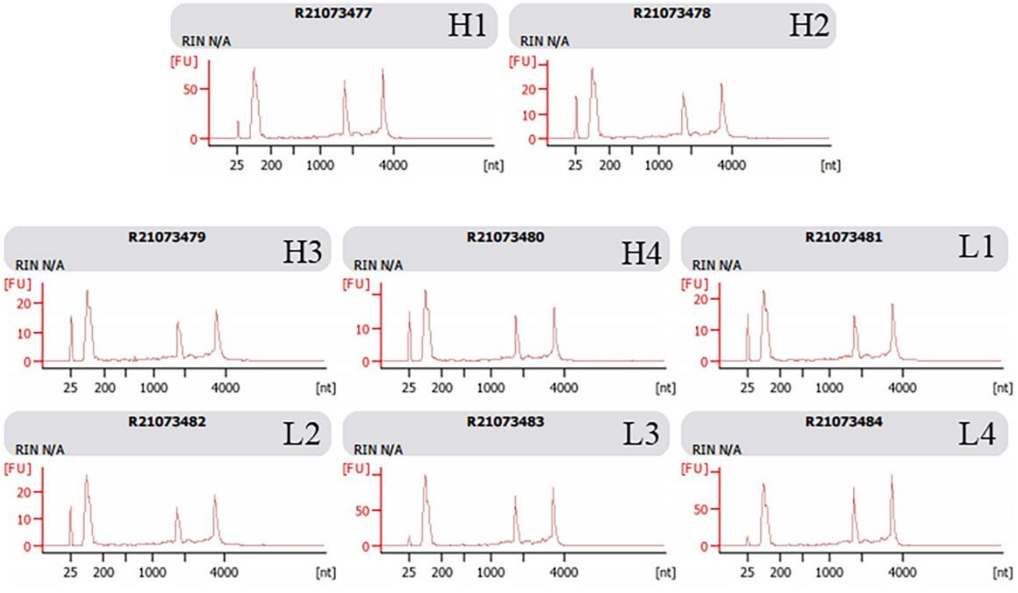

Figure S5. Electropherogram analysis of Total RNA on Agilent 2100 Bioanalyzer.

Note: The RIN value sometimes appears N/A when the Nanodrop 2100 instrument detects the quality of RNA. This phenomenon is relatively common. It did not mean that the RNA has no RIN value, but because the instrument cannot distinguish some peak types for a while, so it is represented by N/A.
